# Glyco-engineered multifunctional hydrogel with bacterial fishing hooks for targeted bacterial eradication and enhanced wound healing

**DOI:** 10.1016/j.mtbio.2025.102174

**Published:** 2025-08-05

**Authors:** Peng Jiang, Shuoyi Zhang, Zheng Tang, Weilan Wang, Haibo Mu, Kaixu Chen

**Affiliations:** a Xinjiang Key Laboratory of Herbivore Nutrition for Meat&Milk, College of Animal Science, Xinjiang Agricultural University, Urumqi, 830052, China; b Xinjiang Key Laboratory of Biological Resources and Genetic Engineering, College of Life Science and Technology, Xinjiang University, Urumqi, 830046, China; c College of Chemistry & Pharmacy, Northwest A&F University, Yangling, 712100, China

**Keywords:** Drug-resistant bacteria, Wound, Multifunctional hydrogel, Photothermal therapy, Black phosphorus

## Abstract

Infection with drug-resistant bacteria in hospitals significantly hinders wound healing, resulting in a high mortality rate among affected patients. In this study, we developed a multifunctional dextran-based hydrogel platform that effectively captures and eliminates carbapenem-resistant *Pseudomonas aeruginosa* (CRPA) from wounds. By grafting glycoligands (galactose and fucose) onto the three-dimensional hydrogel matrix and two-dimensional black phosphorus nanosheets, we substantially enhance the selective capture of CRPA through multivalent carbohydrate-lectin interactions. These glycoligands as hooks not only improve the stability of black phosphorus nanosheets, reducing their susceptibility to degradation, but also enable the hydrogel platform to spontaneously capture bacteria, localizing thermal ablation at the site of infection. This dual-action approach not only enhances bactericidal efficacy but also minimizes adverse effects on surrounding healthy tissue. Furthermore, the hydrogel exhibits multifunctional properties, including biocompatibility, anti-inflammatory activity, hemostasis, adsorption, adhesion, self-healing, and sufficient mechanical performance, ensuring its *in vivo* therapeutic efficacy in a mouse model of CRPA-induced cutaneous infections. This study demonstrates the utilization of glycohooks to optimize the photothermal effects of black phosphorus nanosheets, potentially opening new avenues for precisely synergistic therapies based on photothermal nanomaterials to combat antibiotic-resistant bacteria.

## Highlights

• A multifunctional platform is constructed using glycohook-grafted gel and black phosphorus.
• Glycohooks improve CRPA capture via multivalent carbohydrate-lectin interactions.
• The platform exhibited anti-inflammatory, adhesion, self-healing, and robust mechanics.
• It showed hemostasis, degradability, biocompatibility and toxin adsorption.
• It promoted healing of CRPA-infected wounds.

## Graphical abstract

**Figure:**
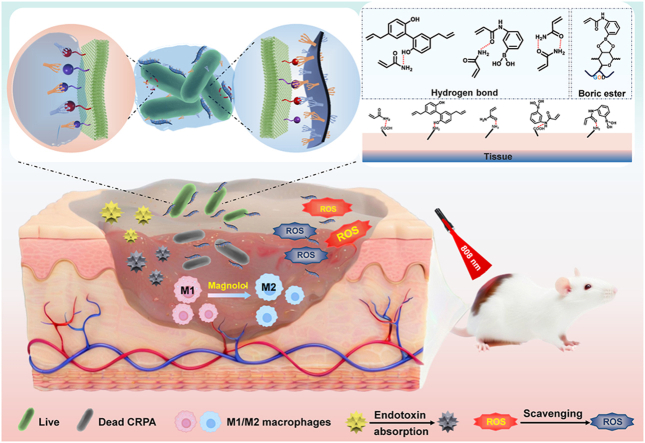

## 1 Introduction

Bacterial infections, especially those caused by drug-resistant strains, result in over ten million deaths annually and cause millions of clinical illnesses worldwide [1]. Treating these infections often requires higher dose or multiple antibiotics, which can accelerate resistance and cause side effects, creating a vicious circle [2]. By 2050, bacterial infections could cause ten million deaths per year if efforts to curb resistance or develop new antibiotics are not intensified [3]. Given the high costs and slow progress in developing new antibiotic, functional nanomaterials with potent antibacterial effects have emerged as potential alternatives.

Nanomaterials with photothermal effects, which kill bacteria through heat ablation without inducing resistance, have gained significant interest. However, rude photothermal therapy (PTT) can harm normal tissues due to overheating from aggregation in physiological environments, leading to potential toxicity [4]. Additionally, these nanomaterials often fail to interact effectively with bacteria, reducing PTT efficiency. To address this, positively charged polymers such as phenylboronic acid, chitosan, or polylysine are used to modify PTT agents for electrostatic attraction of bacteria [[5], [6], [7], [8]]. Additionally, nanohooks with physical pegs have been constructed to capture bacteria [9]. However, these methods based on van der Waals forces, electrostatic forces, hydrophobic interactions, or physical interaction lack specificity because they cannot distinguish bacteria from normal tissue. Bacteria use lectins, carbohydrate-binding proteins, to specifically interact with saccharides on host tissues. For example, *P. aeruginosa* uses galactose-specific lectin A (LecA) and fucose-specific lectin B (LecB), while *E. coli* uses mannose-binding lectin to adhere to host cells [10,11]. These receptors offer precise targets for multivalent ligands [12,13], leading to the development of glycoconjugates that modify PTT agents like graphene, Au nanorods, or molybdenum disulfide to target specific bacteria and enhance PTT efficacy [8,10,14]. However, these glyco-modified nanomaterials remain in powder form, making them unsuitable for biomedical applications [[15], [16], [17]].

As a rising star in two-dimensional nanomaterials, black phosphorus (BP) nanosheets have gained attention for biomedical applications due to their superior properties: high photothermal conversion efficiency, large surface-to-volume ratio, excellent biocompatibility, and good biodegradability [18]. Their photothermal performance makes BP nanosheets ideal for photothermal therapy (PTT), where they convert light energy into heat to destroy bacteria or cancer cells [[19], [20], [21]]. However, BP's instability under ambient conditions, caused by its reactive electrons, limits its practical application [6,22]. This instability necessitates measures to passivate the lone-pair electrons of phosphorus atoms. Moreover, BP nanosheets' inability to directly interact with bacteria reduces PTT efficiency. An additional carrier is needed to enhance BP nanosheets' dispersion and improve PTT efficacy under NIR irradiation.

Recently, hydrogels have garnered considerable attention in the biomedical field owing to their high porosity, soft consistency, and capacity to absorb excess tissue exudate, while maintaining wound moisture. Their tunable chemical compositions enable a wide range of structures and properties [[23], [24], [25]]. Research efforts have focused on integrating biological elements into hydrogel design to modulate local wound microenvironments and promote healing processes through mechanisms such as scavenging reactive oxygen species (ROS), photothermal/photodynamic therapy, and immune cell regulation [26,27]. The inherent three-dimensional structure of hydrogels provides ideal multiple branching sites for saccharides modification. Given that saccharide-lectin binding interactions are typically weak on a per-molecular basis, these interactions can be significantly amplified using glycodendritic architectures based on multivalent binding, thereby improving bacterial recognition, adhesion and promoting antibacterial effects [[28], [29], [30]].

**Scheme 1:**
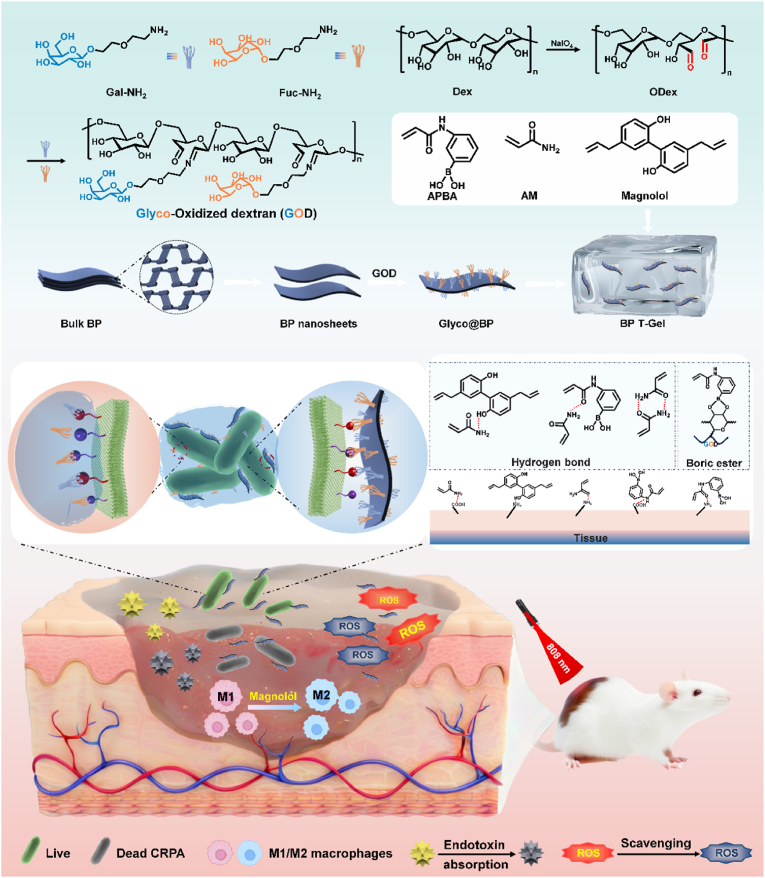
Schematics for preparation and architecture of BP-T-Gel. The envisioned paradigm of BP-T-Gel to capture and eliminate bacteria, adsorb toxin, anti-inflammatory, and promote wound healing.

In this work, we constructed a multifunctional therapeutic hydrogel platform that integrates specific bacterial capture and BP nanosheets induced PTT (Scheme 1). This hydrogel platform features glyco-grafted oxidized dextran hydrogels with multimodal glycodendritic architectures designed to enhance the recognition and capture of bacteria. Additionally, it serves as a carrier for loading BP-based multivalent glycosheets. The porous network of the hydrogel not only facilitates the dispersion of BP glycosheets for controlled heat release but also protects BP from chemical degradation, thereby enhancing its environmental stability. Moreover, the hydrogel exhibits multifunctional properties such as excellent biocompatibility, anti-inflammatory activity, hemostasis, adsorbability, adhesion, self-healing, and sufficient mechanical performance, ensuring the feasibility of this platform for future clinical applications. This strategy presented a novel proof-of-concept framework, demonstrating the application of glycohooks to enhance the PTT efficacy of BP nanosheets. It offered experimental evidence supporting the development of precisely coordinated synergistic therapies based on photothermal nanomaterials to combat antibiotic-resistant bacteria.

## 2 Materials and methods

### 2.1 Materials

D-galactose (Gal), L-fucose (Fuc), Dextran (Mw 12 kDa), acrylamide (AM), acrylamidophenylboronic acid (APBA), 5,5′-diallyl-2,2′-biphenyldiol (magnolol, Mag), 3-(NH_4_)_2_S_2_O_8_ (ammonium persulphate, APS), N,N′-methylenebisacrylamide (MBA), 2-(2-chloroethoxy)ethanol and sodium azide were purchased from Macklin (Shanghai, China). Black phosphorus (BP) was purchased from Hangzhou Nano-Mall Technology Co. Ltd (Hangzhou, China). Tryptic soy broth and nutrient agar were purchased from AOBOX (Beijing, China). Methicillin resistant *Staphylococcus aureus* (MRSA, ATCC 43300), *P*. *aeruginosa* (PA, ATCC 27853) and carbapenem-resistant *P*. *aeruginosa* (CRPA, SHMCC D25221) were purchased from BeNa (Beijing, China). Mouse fibroblast cell (L929) was purchased from Gaining Biological (Shanghai, China). Dulbecco's modified eagle medium (DMEM), penicillin and streptomycin were purchased from Gibco (NY, USA). Fetal bovine serum (FBS), 3-(4,5-dimethylthiazol-2-yl)-2, 5-diphenyltetrazolium bromide (MTT) were purchased from Solarbio (Beijing, China). Propidiumlodide (PI) was purchased from Keygen biotech (Nanjing, China). SYTO 9 green fluorescent nucleic acid stain was purchased from Macklin (Shanghai, China). Annexin V-FITC/PI apoptosis detection kit and 4′,6-diamidino-2-phenylindole (DAPI) was purchased from BestBio (Shanghai, China). All other chemicals were analytical grade and used as received without further purification.

### 2.2 Preparation of BP nanosheets

BP nanosheets were prepared by using a non-organic solvent modified liquid exfoliation of BP powders as previously reported [31]. In brief, 100 mg of BP powders was dispersed in 100 mL ultrapure water. For reducing the oxidation during the exfoliation procedure, the suspension bubbled with argon to eliminate the dissolved oxygen molecules. The mixture solution was tip-sonicated for 12 h (Power: 240 W, On/Off cycle: 10 s/5 s) in ice bath. In order to get rid of the nonexfoliated, the resulting brown suspension was centrifuged at 3000 rpm for 10 min to collect the supernatant containing BP nanosheets, then further concentrated at 12000 rpm (4 °C) for 10 min. The precipitate was re-suspended with a certain amount of ultrapure water to determine the concentration of BP nanosheets by the absorbance at 489 nm using a spectrophotometer (PERLONG, DNM-9612, Beijing, China). Afterward, large amounts of the remaining BP nanosheets were stored under 4 °C for further use.

### 2.3 Synthesis of glyco ligands grafted oxidized dextran (GOD)

The oxidized dextran was prepared by oxidizing dextran with sodium periodate [32]. To synthesize glyco ligands grafted oxidized dextran (GOD), 1.2 mL of oxidized dextran (60 mg/mL), 100 μL of Gal-NH_2_ (30 mg/mL) and 100 μL of Fuc-NH_2_ (30 mg/mL) were mixed at pH 9 under stirring for 10 min.

### 2.4 Preparation of glyco@BP nanosheets

10 mL of deionized water containing bare BP nanosheets (1 mg), Gal-NH_2_ (10 mg) and Fuc-NH_2_ (10 mg) was sonicated for 30 min and stirred overnight. Then, the suspension was centrifuged at 3000 rpm for 10 min to collect the supernatant containing glyco@BP nanosheets, further concentrated at 12000 rpm for 10 min (4 °C), and washed three times with PBS. The concentration of glyco@BP was determined by absorbance at 489 nm.

### 2.5 Preparation of the AM, Gel, T-Gel and BP-T-Gel hydrogels

The hydrogel was fabricated via a free-radical polymerization method. For BP-T-Gel, 2 mL of deionized water containing 100 μg/mL of glyco@BP (50 μg/mL of Gal-NH_2_, and 50 μg/mL of Fuc-NH_2_), AM (2 M), APBA (10, 15, 20 mM), magnolol (10, 15, 20 mM) and GOD (10, 15, 20 mM) were mixed with APS (0.14 mM). The final relative molar concentrations of AM:APBA:magnolol:GOD were 200:1:1:1, 200:1.5:1.5:1.5 and 200:2:2:2, respectively. The mixed solution was cultured at 60 °C for 120 min in a water bath and then the deep grey BP-T-Gel was obtained. Similarly, AM, APBA, magnolol and GOD were mixed with APS for the pink T-Gel preparation. AM, APBA, magnolol and oxidized dextran were mixed with APS for the oyster-white Gel. Thereafter, the as-prepared hydrogels were immersed in deionized water for two days to remove the unreacted impurities, and finally subjected to freeze drying to get the dry hydrogels.

### 2.6 Photothermal performance

An 808 nm NIR laser (NINGBOYUANMING, LSR808NL-2W, Ningbo, China) was utilized to induce the photothermal performance of BP-T-Gel. In general, Gel or BP-T-Gel were cut into small pieces (diameter 10 mm, thickness 2 mm), and irradiation by 808 nm NIR laser (0.5, 1, 1.5 W/cm^2^) for 9 min. Equal volume of PBS was used as control. During irradiation, the temperature was recorded every 30 s with a thermal imaging camera (UNI-T, UTi220A, Qingdao, China). The photothermal performance of the BP nanosheets and glyco@BP nanosheets were investigated using the same method. Briefly, 1.5 mL of BP nanosheets and glyco@BP nanosheets dispersion (100 μg/mL) in PBS were added into plate. Subsequently, the 808 nm NIR laser (0.5, 1, 1.5 W/cm^2^) was focused on the suspensions for 30 min. Then, glyco@BP powder (500 μg) was irradiated with 808 nm NIR laser (0.5, 1, 1.5 W/cm^2^) for 20 s.

The photothermal stability of glyco@BP nanosheets was investigated by irradiating glyco@BP powder (500 μg) with an 808 nm NIR laser (1 W/cm^2^) for four on-off cycles.

### 2.7 Bacterial capture capacity

Carbapenem-resistant *Pseudomonas aeruginosa* (CRPA), PA and MRSA were used to evaluate the capture capacity of glyco@BP or BP-T-Gel [10]. First, bacteria (10^8^ CFU/mL) were incubated with the glyco@BP nanosheets or BP-T-Gel granule for 1 h, the bacteria were adhered to glyco@BP nanosheets and BP-T-Gel granule, respectively. Afterward, diamidino-2-phenylindole (DAPI, 10 μL, 10 μg/mL) fluorescent dyes were introduced to 200 μL of the above bacteria solution and incubated in the dark for 15 min. The bacteria cultured in PBS was used as controls. Images were acquired using fluorescence microscope (Olympus BX53) and processed by cellSens Entry software. The capture ability was also measured by determining the optical density at 600 nm of the medium. In general, glyco@BP nanosheets (50–150 μg/mL) or BP-T-Gel granule (100 mg) were incubated with 1 mL of bacteria in PBS (0.6 OD_600_) at 37 °C. At regular intervals, the OD_600_ value of the suspension was recorded by microplate reader (PERLONG, DNM-9612, Beijing, China). The hydrogel granule was subjected to SEM imaging as following morphological characterization.

### 2.8 Antibacterial tests

The *in vitro* antibacterial activity of BP-T-Gel was tested by a spread plate method [27]. The gel was cut into cylinders pieces (diameter 10 mm, thickness 2 mm), sterilized by UV irradiation and presoaked in PBS for 1 h to reach absorption equilibrium. Then the pieces were immersed in 1 mL of bacteria suspension (10^6^ CFU/mL) and irradiated with 808 nm NIR laser for 15 min (1 W/cm^2^). PBS treatment was control. After then, the hydrogel pieces were removed after squeezing out the bacterial suspensions. The bacteria suspension (40 μL) was cultured on TSB agar at 37 °C for 24 h for photographs, or 10-fold diluted and cultured for the colony-forming units (CFU) count. The relative bacterial viability was determined using the equation: Bacterial viability (%) = N_t_/N_c_ × 100 %, where Nt and Nc represented the number of bacterial colonies formed in the treatment group and the control group, respectively.

For endotoxin adsorption, 10 mg of hydrogel piece was incubating in 2 mL of endotoxin solution (1 EU/mL) for 30 min, then the concentrations of endotoxin in the supernatant was quantified using an endotoxin detection kit from Genscript (Nanjing, China).

### 2.9 Hemostatic evaluation experiments

The hemostatic ability of hydrogels was evaluated using a mouse-tail amputation model as previous [33]. After cutting 30 % length of mouse tail and exposed in air for 15 s, the wound was quickly wrapped with gauze or hydrogels. The weight and time of blood loss were all recorded to evaluate the hemostatic performance.

For the blood clotting test, whole blood containing citrate dextrose were used [33]. Primarily, 100 μL of recalcified blood containing 20 mM CaCl_2_ was dropped on hydrogels pieces (diameter 8 mm). After incubation for 20 min at 37 °C, deionized water (1 mL) was carefully added to disperse the unattached blood without breaking the clot. The optical density at 540 nm of the blood dispersion was measured. 100 μL of blood incubated with 1 mL water served as control. The blood clotting index (BCI) was calculated as equation: BCI (%) = (I_s_/I_r_) × 100 %, where I_s_ and I_r_ were the optical densities of sample group and control group respectively.

Blood cell adhesion on the hydrogels was investigated by dropping the whole blood onto the gauze or hydrogels, and incubated at 37 °C for 5 min. After washing off the free blood cells, the gauze or hydrogel samples were immobilized, dehydrated, and subjected to SEM analysis as described previously [34].

### 2.10 *In vivo* wound healing experiment

Female Kunming mice (5-week-old, ∼25 g) were purchased Tengxin Experimental Animal Co., Ltd. All animal procedures complied with the ARRIVE guidelines 2.0 (Animal Research: Reporting *In Vivo* Experiments) [35], and approved by the Northwest A&F University Animal Care Committee (NWAFU-314020038). As a typical wound infection model, a round wound with a diameter of 10 mm was created on the back of each mouse, and 10 μL of CRPA suspension (10^8^ CFU/mL) was immediately smeared on the wounds. Then covering the wound with medical tape for 24 h to establish the bacteria-infected wound model. Then the mice were randomly divided into four groups (9 mice per group) for different treatment: medical tape, Gel, BP-T-Gel and BP-T-Gel + NIR irradiation (808 nm NIR laser irradiation for 4 min). The wound in the control group was bandaged with medical tape and the wounds in other groups were dressed with different hydrogels pieces. Then a medical tape was used to cover and protect the hydrogels from mice chew. The wound area was recorded and photographed. Meanwhile, the bacteria in the infected tissue were counted at indicated day by tissue homogenate of three mouse in every group. 9 days after treatments, all mice were executed by cervical dislocation, the wound skin tissues were harvested for H&E and Masson staining. The main organs tissues (kidney, heart, spleen, lung, liver, and wound skin) were also harvested for histology examination.

### 2.11 Statistical analysis

Statistical analysis and graphing were performed using GraphPad Prism 8.0 (GraphPad Software, San Diego, USA). Quantitative results are reported as mean values with standard deviations from three replicates. A two-tailed Student's t-test was used for comparing two groups, and one-way ANOVA for multiple groups. *p* < 0.05 was considered significant.

## 3 Results and discussion

### 3.1 Construction and characterization of multivalent glycosheets

NH_2_-tailed glycosides, including Gal-NH_2_ and Fuc-NH_2_, were successfully synthesized through a series of three-step reactions (Scheme S1). The structures of the resulting products were rigorously characterized using HNMR spectroscopy (Fig. S1 and S2).

**Fig. 1:**
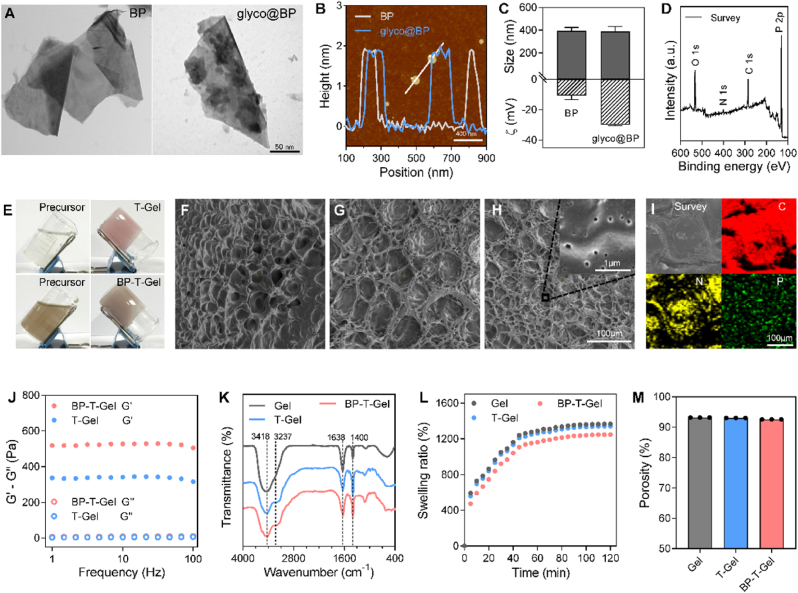
Preparation and characterization of BP-T-Gel. (A) TEM image of BP nanosheets and glyco@BP. (B) AFM image and the corresponding thickness profiles of BP nanosheets and glyco@BP. (C) The size and Zeta potential of BP nanosheets and glyco@BP. (D) XPS spectra of glyco@BP: full scan survey of all elements. (E) Photographs of the precursor solution and resultant T-Gel and BP-T-Gel. Gel was prepared from AM with APBA, magnolol and OD. T-Gel was prepared from AM with APBA, magnolol, and GOD. BP-T-Gel was prepared from AM with APBA, magnolol, GOD and glyco@BP. SEM image of Gel (F), T-Gel (G) and BP-T-Gel (H). (I) EDS element mapping of C, N, and P in BP-T-Gel. (J) Rheology properties of T-Gel and BP-T-Gel with oscillatory frequency sweep by 0.1 % strain. (K) FT-IR spectra of the as-prepared Gel, T-Gel and BP-T-Gel. The swelling ratio (L) and porosity (M) of Gel, T-Gel and BP-T-Gel.

BP nanosheets were synthesized via liquid exfoliation of bulk BP using oxygen-free water as the solvent to prevent excessive oxidation and ensure a pristine surface. Transmission electron microscopy (TEM) revealed that the lateral dimensions of the BP nanosheets were approximately 200 nm, exhibiting typical two-dimensional flake morphology (Fig. 1A). High-resolution TEM (HR-TEM) images confirmed the lattice spacing to be 0.28 nm (Fig. S3A), consistent with the known lattice parameters of BP [36]. Atomic force microscopy (AFM) measurements (Fig. 1B) indicated a thickness of approximately 2 nm for the BP nanosheets, verifying the successful preparation of ultrathin BP nanosheets. Subsequently, NH_2_-glyco was immobilized on the surface of BP nanosheets, resulting in the formation of glyco@BP. TEM and AFM analyses (Fig. 1A and B) demonstrated that surface functionalization did not significantly alter the sheet-like morphology or size of the material. After coating with glyco-NH_2_, the zeta potential of BP shifted markedly from −10.72 mV to −29.72 mV (Fig. 1C). Dynamic light scattering (DLS) analysis showed that the hydrodynamic diameter of glyco@BP was approximately 390 nm, comparable to that of bare BP nanosheets (Fig. 1C).

FTIR spectra in Fig. S3B revealed the emergence of new absorption bands at approximately 2900 cm^−1^ (attributed to C-H vibrations in the ethylene glycol segment) and 1100 cm^−1^ (corresponding to C-O stretching vibrations in the NH_2_-glyco structure) upon coating NH_2_-glyco onto BP nanosheets [31]. Scanning TEM-energy-dispersive X-ray images (Fig. S3E) illustrated the homogeneous distribution of four distinct elements across the entire nanosheet surface. The P element originated from BP, whereas O, N, and C elements were attributed to the presence of NH_2_-glyco. X-ray photoelectron spectroscopy (XPS) was utilized to further validate the chemical composition of glyco@BP. As depicted in Fig. 1D and Fig. S3D, peaks at 538.9 eV (O1s), 399.1 eV (N1s), and 284.0 eV (C1s) were identified. Additionally, peaks at 129.1 eV and 130.1 eV corresponded to the P 2p3/2 and P2p1/2 orbitals in the P 2p spectrum, respectively. A weak sub-band at 132.2 eV was assigned to partially oxidized phosphorus (i.e., P_x_O_y_), indicating minor degradation of BP nanosheets. This suggests that the NH_2_-glyco coating effectively mitigated oxidation of BP nanosheets. UV–vis absorbance spectra of glyco@BP exhibited no significant changes compared to bare BP, confirming that the optical properties of BP remained unaffected after glyco-NH_2_ modification (Fig. S3C). Collectively, these results robustly demonstrated the successful surface modification of BP NSs by glyco-NH_2_.

The stability of nanocomposites is critical for a theranostic drug delivery platform. UV–vis spectroscopy was employed to assess the absorbance of BP and glyco@BP NSs under ambient conditions. After 20 days of exposure, the absorbance intensity of glyco@BP aqueous dispersions decreased only marginally (Fig. S4A), indicating its superior stability. In contrast, the absorbance intensity of bare BP nanosheets decreased sharply by 30 % over the same period, highlighting the enhanced stability of glyco@BP. To evaluate photothermal stability, temperature changes were monitored using an infrared thermal imaging camera during NIR irradiation (808 nm, 30 min). As shown in Fig. S4B, the temperature of bare BP suspension increased to 47.8 °C after irradiation. However, after 20 days of storage, the temperature of the bare BP suspension under irradiation dropped to 35.2 °C, indicating a significant reduction in photothermal properties. In comparison, the temperature of glyco@BP suspension reached 42.8 °C after 20 days of storage, significantly higher than that of bare BP under identical conditions. To further investigate the degradation behavior, morphological changes were examined via TEM. For bare BP nanosheets (Fig. S4C), numerous degradation holes appeared on the surface after 20 days of exposure to water and air. This phenomenon can be attributed to the oxidation of BP's van der Waals surface by pure oxygen and pre-existing defects by water [6,37]. By contrast, glyco@BP exhibited fewer bubbles and retained longer strips due to its enhanced stability. In addition, the degradation of glyco@BP was also tested by measuring the phosphorus content [38]. As shown in Fig. S5A and S5B, the total phosphorus content in the supernatant of the glyco@BP dispersion stored at room temperature for 20 days was approximately 10.45 μg/mL. In contrast, the phosphorus content in the BP group was more than twice that of the glyco@BP group, reaching approximately 22.59 μg/mL. All these findings strongly suggest that glyco-NH_2_ modification can protect BP nanosheets from oxidative degradation [6], thereby enhancing their stability and biocompatibility in physiological media.

The fabrication process of the glycohook-equipped hydrogel (T-Gel) is illustrated in Scheme 1. Initially, dextran was oxidized by sodium periodate to form oxidized dextran (OD), which contains multiple aldehyde groups. The oxidation substitution degree of OD was determined to 39.96 % using a hydroxylamine hydrochloride titration method [39,40]. This OD subsequently reacted with amino groups from glyco-NH_2_ to produce glyco-oxidized dextran (GOD). The glycosyl ligand substitution in the resulted GOD was 30.21 % (Fig. S6A). In the presence of GOD, T-Gel was synthesized via copolymerization of acrylamide (AM), aminophenylboronic acid (APBA), and magnolol (Fig. 1E). Within the T-Gel matrix, various physical interactions such as hydrogen bonding, Schiff base formation, and high affinity between boronic acids and diols confer the hydrogel with excellent mechanical flexibility and elasticity. This is particularly noteworthy given that chemically crosslinked AM hydrogels typically exhibit robustness and rigidity. Indeed, stable hydrogels could hardly be formed without APBA or GOD (Fig. S6B). Rheological analysis in Fig. S6C revealed that the hydrogel (200:2) exhibited the highest storage modulus of approximately 900 Pa. As the content of APBA/magnolol/GOD decreased, the storage modulus reduced to about 300 Pa for the hydrogel (200:1.5), indicating that an optimal ratio is essential for achieving elastic deformation. SEM images in Fig. 1G showed no obvious porous microstructures, likely due to pore shrinkage during freeze-drying, suggesting a high crosslink density in the hydrogel. However, after swelling in water, the hydrogel displayed a three-dimensional porous microstructure with pore sizes ranging from tens to hundreds of micrometers, potentially enhancing air permeability. FTIR spectra were measured to confirm the successful fabrication of T-Gel. As shown in Fig. 1K, the peak at 3418 cm^−1^ was attributed to the N-H stretching vibration of the acrylamide (AM) monomer, while the peak at 1638 cm^−1^ was assigned to the C=C stretching vibration of the benzene ring. The peak at 1400 cm^−1^ was attributed to the B-O stretching vibration. Notably, the O-H stretching vibration peak at 3237 cm^−1^ was observed for both T-Gel and BP-T-Gel, indicating the incorporation of GOD into the hydrogels [41].

BP-T-Gel was synthesized by incorporating glyco@BP nanosheets into the precursor solution prior to copolymerization (Fig. 1E). Compared to T-Gel, BP-T-Gel exhibited a similarly dense structure but with a rougher surface (Fig. 1H), which can be attributed to the presence of glyco@BP nanosheets. Notably, all hydrogels displayed a porous structure after swelling in water. Elemental mapping images in Fig. 1I revealed uniform distributions of P, C, and N elements throughout the hydrogel, confirming the successful preparation of BP-T-Gel. Dynamic rheological measurements of BP-T-Gel and T-Gel indicated that the storage modulus exceeded the loss modulus across the entire frequency range (Fig. 1J), demonstrating clear viscoelastic behavior and confirming the formation of a stable hydrogel network. Additionally, flow dynamics at various temperatures showed consistent dynamic moduli (Fig. S6D), suggesting good thermal stability of the prepared hydrogel.

The management of wound exudate represents a significant advantage of hydrogel dressings. The equilibrated swelling ratio is commonly utilized to assess the exudate absorption capacity of hydrogels. Specifically, the swelling ratio of BP-T-Gel reached 10-fold within 40 min (Fig. 1L), which was slightly lower than that of T-Gel (11-fold). This difference may be attributed to the incorporation of BP slightly affecting the interchain hydrogen bonding of the polymer chains, suggesting that nanosheet loading has minimal impact on swelling capacity. The degradation performance of hydrogel was evaluated by monitoring its weight loss over time (Fig. S7A). BP-T-Gel and T-Gel exhibited gradual decreases in mass over time and showed a similarly remaining mass of about 79 % after 9 days in PBS. This observation indicates a slow degradation behaviour under *in vitro* conditions. Additionally, porosity was evaluated using an alcohol displacement method. As illustrated in Fig. 1M, all hydrogels exhibited consistent porosity (∼92 %). The high porosity of the hydrogels not only enhances the absorption of wound exudates but also promotes gaseous exchange, both of which are beneficial for wound healing.

### 3.2 Mechanical and photothermal properties of hydrogel

**Fig. 2:**
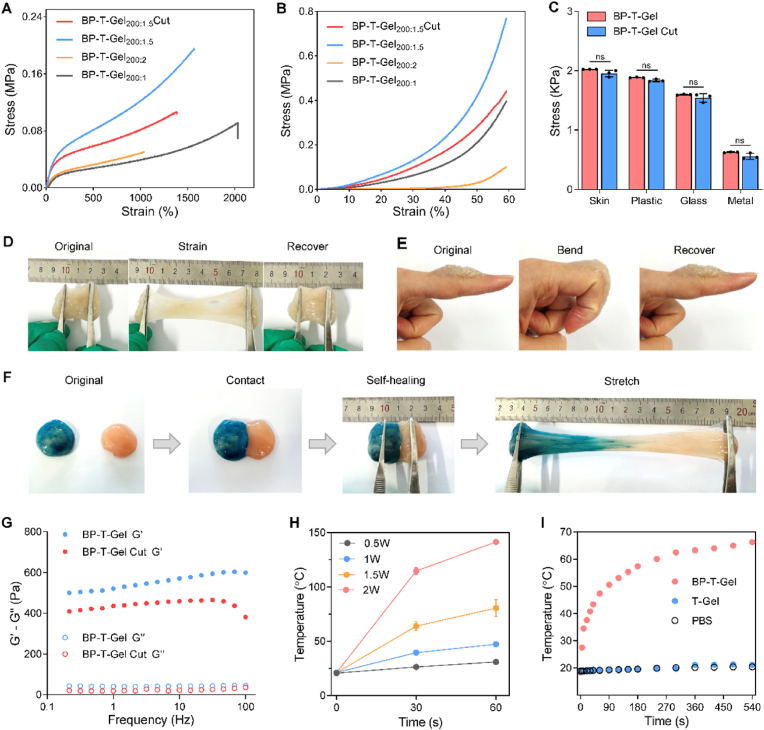
Mechanical and photothermal properties of BP-T-Gel. (A) The tensile stress-strain curves of BP-T-Gel with different APBA/magnolol/GOD concentrations. (B) The compressive stress-strain curves of BP-T-Gel. (C) Adhesion strengths of BP-T-Gel and self-healed BP-T-Gel (BP-T-Gel Cut) to skin tissues, plastic, glass and metal (n = 3). (D) BP-T-Gel withstood large stretching with external force. (E) BP-T-Gel adhered to the knuckle and bent easily. Author Peng Jiang was the subject of this experiment, and an informed consent was obtained. (F) BP-T-Gel healed after contact at room temperature for 10 min and the self-healed hydrogel was able to withstand external tension. Blue was BP-T-gel with methylene blue, Pink was T-Gel. (G) Rheology properties of BP-T-Gel and BP-T-Gel Cut with oscillatory frequency sweep by 0.1 % strain. (H) The temperature curves of BP-T-Gel with different NIR power. (I) Temperature curves of BP-T-Gel under NIR irradiation with a power of 1 W/cm^2^.

The hydrogels formed by multiple crosslinks typically exhibit superior mechanical properties, including excellent flexibility and recoverability. Tension and compression tests were conducted to evaluate the mechanical performance of BP-T-Gel. As shown in Fig. 2A, BP-T-Gel with an optimal APBA/magnolol/GOD content of 200:1.5 demonstrated the highest tensile fracture strain of 1560 % and a tensile fracture stress of 194 kPa, indicating its exceptional stretchability. Notably, the hydrogel piece can endure significant deformation without sustaining damage and rapidly return to its original state (Fig. 2D). The compression test results presented in Fig. 2B revealed that the maximum compression strength was 770 kPa at 60 % strain with APBA/magnolol/GOD content of 200:1.5. The remarkable elasticity and resilience are attributed to the synergistic effects of physical crosslinking via host-guest interactions and hydrogen bonding. Furthermore, the tension curves of BP-T-Gel with different water absorption percentages were tested in Fig. S7B. The tensile strength decreased with the increase of water content, but the elongation increased with the increase of water content, which might be due to the expansion and stretching of the three-dimensional structure of the hydrogel.

Dressings with excellent tissue adhesive strength can gently cover the wound site and create a nurturing microenvironment that promotes tissue regeneration [42]. As shown in Fig. 2C and Fig. S7C, the BP-T-Gel hydrogel patch adhered effectively to various surfaces, including skin, plastic, glass, and metal, with the adhesive stress of 2.02, 1.88, 1.60 and 0.63 kPa respectively. When applied to the author's finger, the hydrogel allowed for natural bending without any restrictions (Fig. 2E). In practical use, these dressings are especially suitable for areas like joints or regions that experience frequent movement or localized stress [43]. Additionally, as demonstrated in Fig. 2F, the BP-T-Gel exhibited remarkable self-healing properties. Two separated pieces of hydrogel fused together after just 10 min of contact and could withstand significant stretching. The self-healed hydrogel (BP-T-Gel Cut) showed a decreased tensile fracture strain to 1380 % and a reduced tensile fracture stress to 106 kPa (Fig. 2A), while the maximum compression strength of self-healed BP-T-Gel were reduced to 443 kPa at 60 % strain. We further assessed the rheological recovery in Fig. 2G, although the self-healed BP-T-Gel exhibited some loss in storage modulus, it retained a relatively high rheological stress. Moreover, the self-healed BP-T-Ge showed a comparable adhesive strength to BP-T-Gel (Fig. 2C). These results indicate that the self-healed hydrogels still retain most of their mechanical properties. This self-healing ability is primarily due to the rearrangement of boronate ester bonds. For real-world applications, this rapid self-healing ensures that even if the dressing breaks due to normal body movements or local stress, it can quickly restore its integrity, preventing external infections and maintaining a sterile environment for wound healing [34]. These findings highlight the hydrogel's superior mechanical strength, strong tissue adhesion, and efficient self-healing capabilities, making it ideal for covering wounds, enduring various deformations, and preventing secondary harm during use.

Photothermal therapy is recognized as an effective, antibiotic-free, minimally invasive approach for treating bacterial infections, particularly those caused by drug-resistant bacteria. Temperature plays a crucial role in photothermal therapy (PTT). Numerous studies have highlighted the exceptional NIR-responsive photothermal activity of BP nanosheets, which have been successfully applied in cancer treatment, wound healing, and rheumatoid arthritis management. As shown in Fig. S8A, the temperature of BP nanosheet suspensions increased steadily with irradiation time, and glyco@BP demonstrated a similar temperature curve to that of BP NS. The temperature of glyco-BP powder was notably higher than that in PBS and positively correlated with laser power (Fig. S8B). Moreover, glyco@BP exhibited excellent photothermal stability, with no significant changes observed in the maximum temperature (Fig. S8C). These results confirm that the photothermal properties of BP remain largely unaffected by glyco modification, suggesting great potential for glyco@BP in photothermal antibacterial therapy.

Next, we also explored the photothermal capabilities of the hydrogels. As shown in Fig. 2H, the temperature of BP-T-Gel increased significantly with higher laser power. While elevated temperatures can help prevent infections, they may also pose risks to the skin. Therefore, we carefully set the laser power to 1 W/cm^2^ to ensure safety and effectiveness. The photothermal images and corresponding temperature curves in Fig. 2I and Fig. S8D demonstrate that the temperature of BP-T-Gel rose quickly under NIR irradiation. In contrast, there were no noticeable changes in the Gel or PBS under the same conditions. After 2 min of irradiation, BP-T-Gel reached a temperature of 53.1 °C, which is sufficiently high to inactivate bacteria through mechanisms such as membrane disruption and damage to enzymes or proteins.

### 3.3 Bacterial capture

**Fig. 3:**
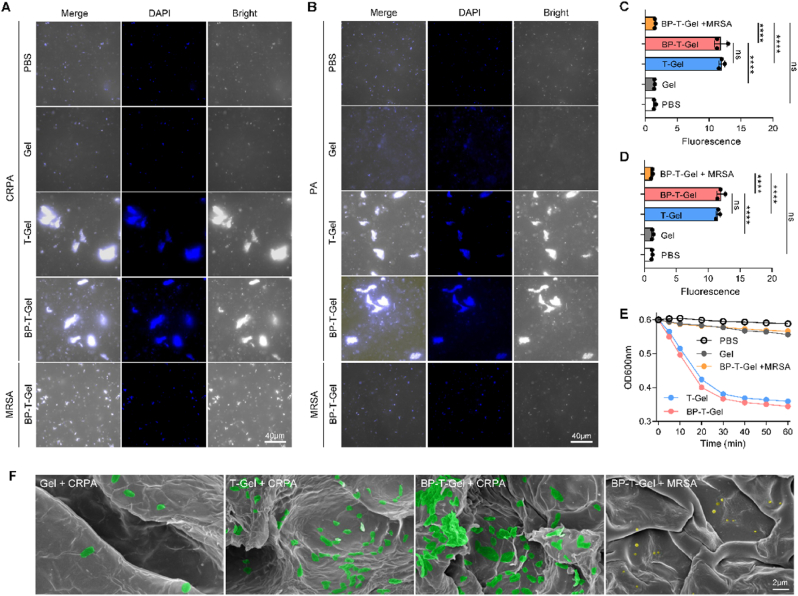
Bacterial capture capacity. Fluorescence images (A and B) and relative fluorescence intensity (C and D) of bacteria incubated with different samples. DAPI (blue) was used to stain bacteria. (E) OD_600_ value of the bacterial supernatant after incubation with different samples. (F) The SEM images of bacteria incubated with hydrogels. CRPA is marked green, and MRSA is yellow.

Fluorescence microscopy was initially utilized to investigate the binding properties of the hydrogel with bacteria. The bacteria were incubated with the hydrogels and subsequently stained with DAPI. As illustrated in Fig. 3A, when cultured with Gel, the CRPA bacteria remained freely dispersed. In contrast, a significant number of bacterial cells formed clusters around T-Gel or BP-T-Gel, exhibiting strong fluorescence (Fig. 3B). Similarly, comparable results were observed in PA (Fig. 3B and D). This glycohook-induced specific bacteria capture was further confirmed by incubating bacteria with glyco@BP nanosheets. As shown in Fig. S9A and S9B, CRPA treated with glyco@BP displayed substantial fluorescence aggregation, while CRPA treated with BP alone or MRSA treated with glyco@BP remained scattered. We observed that when bacteria suspension was incubated with BP or glyco@BP, the OD_600_ value of the supernatant was significant decreased, indicating that glyco@BP could specifically absorb CRPA in a concentration-dependent manner (Fig. S9C). These results indicate that galactose and fucose on BP nanosheets act as glycohooks, enabling glyco@BP-To capture CRPA and PA bacteria through saccharide-lectin binding interactions. Studies have demonstrated that, despite differences in lectin glycosylation patterns between PA and CRPA, these two strains share more than 82 % of identical lectins (only 7 out of 39 lectins differ), with widespread expression of both galactose and fucose lectin receptors observed in each [44].

To investigate the hydrogel's ability to bind and remove bacteria, we measured the OD_600_ value of the bacteria. As shown in Fig. 3E, after a 20 min incubation with T-Gel or BP-T-Gel, the OD_600_ value of CRPA significantly decreased. In contrast, there was almost no change in OD_600_ value even after 1 h of incubation with Gel. Additionally, MRSA incubated with BP-T-Gel showed minimal reduction in OD_600_ value. These results suggest that the glycohooks enable the gel to quickly and specifically bind to CRPA, leading to a lower OD_600_ value. The specific adhesion mechanism involves the binding of LecA and LecB on the surface of CRPA to galactose and fucose, respectively. Further analysis using SEM revealed the interaction between the hydrogel and bacteria. As depicted in Fig. 3C, T-Gel and BP-T-Gel effectively captured more bacteria. The SEM images also showed more CRPA bacteria (green) aggregations in T-Gel and BP-T-Gel, with only a few MRSA bacteria (yellow) scattered in BP-T-Gel (Fig. 3D). Collectively, these findings demonstrate that the prepared hydrogel can efficiently capture bacteria, which would enhance its antibacterial activity.

### 3.4 *In vitro* antibacterial activity

**Fig. 4:**
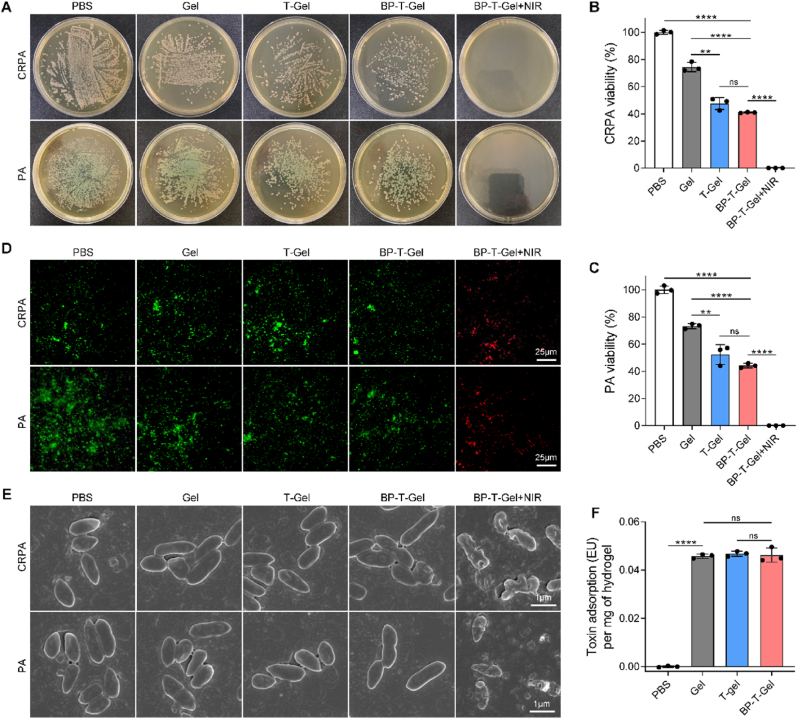
Antibacterial efficacy *in vitro*. Represent photographs of agar plates (A) and corresponding colonies counts (B and C) of bacteria treated with different samples. (D) Live/dead fluorescence images of bacteria treated with different samples. PI (red) was used to label dead bacteria and Syto-9 (green) to live bacteria. (E) SEM images of bacteria treated with different samples. (F) Absorption properties for endotoxin. Data are presented as means ± SD (n = 3). ∗∗*p* < 0.01, ∗∗∗∗*p* < 0.0001. ns, no significant difference.

Considering the remarkable photothermal properties and bacteria-capture capacity of BP-T-Gel, we evaluated its antibacterial effectiveness against CRPA, PA and MRSA *in vitro* (Fig. S10A). As illustrated in Fig. 4A–C, when BP-T-Gel was combined with NIR treatment, it successfully eliminated all bacteria, leaving no survivors on the agar plate. This demonstrates the potent bactericidal activity of BP-T-Gel under NIR irradiation. The bacterial viability significantly decreased when treated with BP-T-Gel or T-Gel alone, primarily due to their excellent bacteria-capture ability. These gels effectively absorbed the bacteria, resulting in a lower count of viable bacteria in the culture medium. Similar results were also observed in MRSA treated with BP-T-Gel or T-Gel (Fig. S10B and C). BP-T-Gel + NIR exhibited a moderate inhibition rate of 50 %, which was mainly attributed to the photothermal effects of BP-T-Gel with NIR irradiation. This was further confirmed by a live/dead cell assay using SYTO 9/PI staining (Fig. 4D). Treatment with BP-T-Gel or T-Gel alone showed no red-stained bacteria, indicating that these hydrogels primarily captured rather than killed the bacteria. In contrast, CRPA or PA treated with BP-T-Gel + NIR were all stained red, showing no survivors, which confirms the powerful inactivating ability of this combination. This is consistent with the viability results shown in Fig. 4A. In contrast, only a subset of the bacterial population exhibited red staining of MRSA in BP-T-Gel + NIR group (Fig. S10D). The difference in bactericidal performance was primarily attributed to the hydrogel's varying capture capabilities toward the different bacterial species. Once PA or CRPA was captured, it came into closer proximity with the hydrogel, thereby enhancing the effectiveness of photothermal action. In contrast, MRSA was rarely captured by BP-T-Gel, resulting in a limited photothermal inactivation effect. SEM was used to investigate morphological changes. As seen in Fig. 4E, the bacterial cell surfaces treated with BP-T-Gel or T-Gel remained intact and smooth, similar to the native live bacterial cells in the PBS group, proving once again that BP-T-Gel or T-Gel did not kill the bacteria. After NIR exposure, however, the bacterial cells treated with BP-T-Gel exhibited severely damaged and ruptured membranes, leading to cell death. In fact, NIR alone or NIR + T-Gel alone is ineffective in eliminating bacteria (Fig. S11), because the brief period of NIR irradiation could not elevate the temperature of PBS or T-Gel significantly (Fig. 2I) and was insufficient to generate adequate heat for bacterial inactivation. Collectively, these results suggest that BP-T-Gel can effectively capture bacteria and subsequently inactivate them through localized overheating induced by NIR. Additionally, all hydrogels efficiently adsorbed endotoxins, even after incorporating glyco@BP (Fig. 4F), which helps reduce local tissue inflammation.

### 3.5 Biocompatibility, cell migration and antioxidant activity

**Fig. 5:**
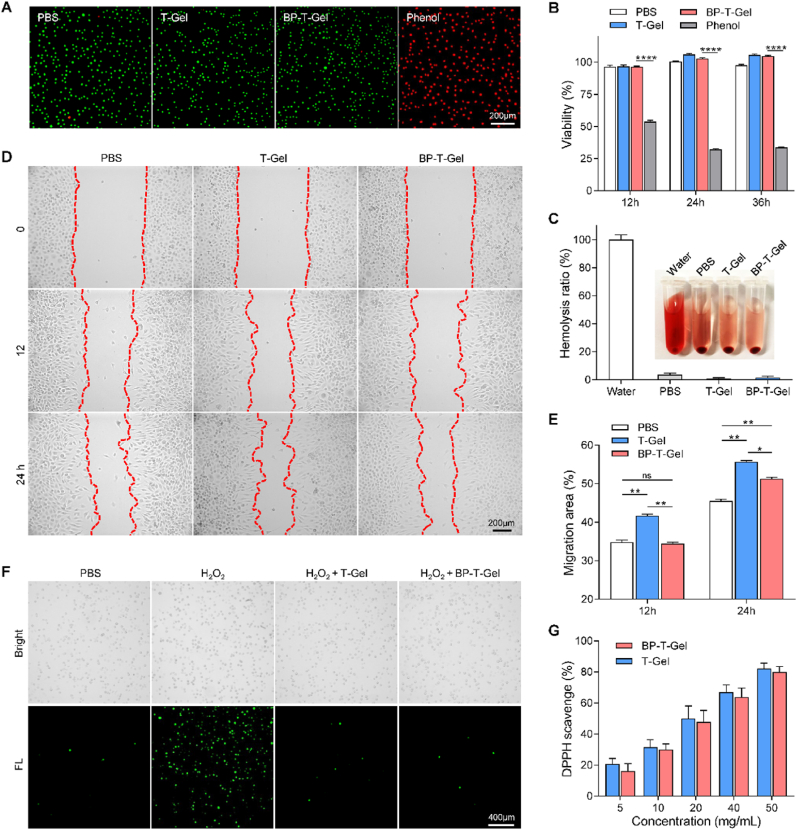
Biocompatibility and biological properties of BP-T-Gel. Live/dead staining (A) and viability (B) of L929 cells treated with different samples. (C) Hemolytic activity of BP-T-Gel. The effect of BP-T-Gel on cell migration of L929 cells: Cell scratch test (D) and migration area (E). (F) Fluorescence images of L929 cells treated with PBS, H_2_O_2_, T-Gel followed by H_2_O_2_, or BP-T-Gel followed by H_2_O_2_. Intracellular ROS was indicated by DCFH-DA. (G) DPPH scavenging capabilities of hydrogels. Data are presented as means ± SD (n = 3). ∗*p* < 0.05, ∗∗*p* < 0.01, ∗∗∗∗*p* < 0.0001. ns, no significant difference.

L929 mouse fibroblasts cells were utilized for biocompatibility evaluation. The cytotoxicity of BP-T-Gel was assessed using Live/dead staining, where live cells were highlighted with green fluorescence via calcein-AM, and dead cells were marked with red fluorescence via propidium iodide (PI). As presented in Fig. 5A, L929 cells treated with the hydrogel exhibited vibrant green fluorescence, indicating they were all alive, similar to those treated with PBS. In contrast, the positive control cells (treated with phenol) appeared predominantly red, indicating significant cell death. After co-culturing for 12–36 h, cells treated with the hydrogel showed excellent cytocompatibility, with cell viability even surpassing that of the PBS group, as confirmed by the Live/dead assay results (Fig. 5B). According to Ref. [45], the degradation products of black phosphorus are mainly phosphates. We also tested the cytotoxicity of the degradation products of glyco@BP from different degradation times. As shown in Fig. S5C and S5D, all the degradation products of glyco@BP exhibited no significant cytotoxicity. Additionally, the phosphate-induced calcification was also investigated. As shown in Fig. S12, the calcium deposition in L929 cells using an Alizarin Red S assay kit [46], both the blank group and the BP-T-Gel treatment group exhibited a relative low level of calcium deposition, with values of 0.115 ± 0.1263 mM and 0.117 ± 0.1324 mM, respectively. This result indicated that the BP-T-Gel does not cause calcium deposition. In fact, many literature have reported the safety of black phosphorus in biomedical engineering applications [17,31,36,[47], [48], [49]]. To further evaluate hemocompatibility, a hemolysis assay was conducted. As illustrated in Fig. 5C, blood samples treated with the hydrogel and PBS displayed a similar appearance, with reddish supernatants and substantial erythrocyte aggregation, unlike the deep red solution with minimal aggregation observed in the water group. Hemolysis ratios were calculated using the water group as the 100 % hemolysis standard, and all hydrogels demonstrated excellent hemocompatibility, with hemolysis ratios below 5 %. During wound healing, cells at the wound edges migrate towards the center to seal the wound. Enhanced cell migration is therefore beneficial for wound repair. As predicted in Fig. 5D and E, compared to the PBS group, the hydrogel promoted cell migration towards the center of the interspace over time, confirming its excellent cytocompatibility and aligning with the MTT results in Fig. 5B. The migration area of BP-T-Gel is smaller than T-Gel, this can be attributed to the fact that there are less magnolol in BP-T-Gel compared to T-Gel at equal weight. Even so, the cell migration areas of both groups are significantly larger than that of PBS group, confirming that BP T-Gel possess a potential for promoting cell migration and tissue regeneration. The above results demonstrate that the hydrogel has good biocompatibility, which supports its potential for further medical applications.

Since the accumulation of excessive reactive oxygen species (ROS) at the wound site can lead to oxidative stress, impaired cellular functions, and damage to proteins, lipids, and DNA, wound dressings that can effectively reduce ROS levels can significantly benefit the wound healing process. Inspired by the presence of magnolol and the dynamic nature of boronate esters in our hydrogel, we evaluated its ROS scavenging ability using a cellular inflammation model. In this model, RAW264.7 cells were treated with H_2_O_2_ to induce oxidative stress, and DCFH-DA, an ROS-sensitive fluorescent dye, was used to monitor intracellular ROS levels. As shown in Fig. 5F, cells treated with H_2_O_2_ exhibited intense green fluorescence, indicating high levels of intracellular ROS. However, this effect was notably reduced when the cells were treated with our hydrogel. Additionally, the hydrogel demonstrated excellent scavenging capacity for DPPH free radicals in a concentration-dependent manner (Fig. 5E). These results suggest that the elaborate hydrogel could be an ideal candidate for addressing excessive ROS in wound dressings, offering significant biocompatibility and therapeutic potential.

### 3.6 Anti-inflammatory activities

**Fig. 6:**
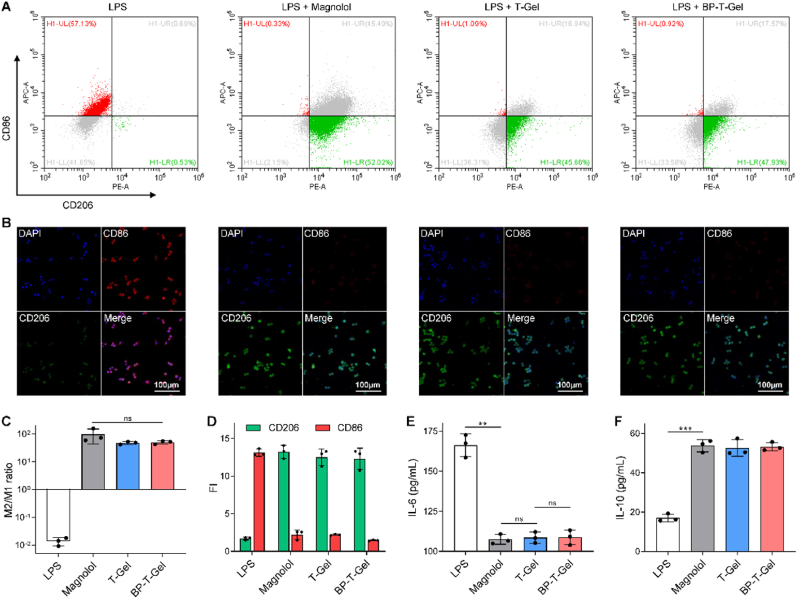
Evaluation of macrophage polarization after the treatment of BP-T-Gel. (A) Representative flow cytometry plots of macrophage cells. Representative images (B), ratio (C) of the M1/M2 levels and fluorescence intensity (D) in the different groups. Expression of IL-6 (E) and IL-10 (F) in RAW264.7 cells induced by LPS in the different groups.

Wound healing is intricately linked to the polarization of macrophages into distinct phenotypes, either pro-inflammatory (M1 phenotype) or anti-inflammatory (M2 phenotype) [50,51]. Notably, it has been shown that Magnolol plays a pivotal role in promoting the transition of M1 macrophages to the M2 phenotype [52]. Through an in-depth analysis employing immunostaining and flow cytometry techniques, we observed that BP-T-Gel effectively facilitated the conversion of LPS-stimulated M1 macrophages into M2 macrophages, as evidenced by an increase in CD206 expression (Fig. 6A and C) and a decrease in CD86 expression (Fig. 6B and D). Additionally, BP-T-Gel treatment significantly downregulated the expression of the pro-inflammatory cytokine IL-6 (Fig. 6E), while simultaneously enhancing the expression of the anti-inflammatory cytokine IL-10 (Fig. 6F). Collectively, these results provide strong evidence for the anti-inflammatory properties of BP-T-Gel patches.

### 3.7 Hemostatic performance

**Fig. 7:**
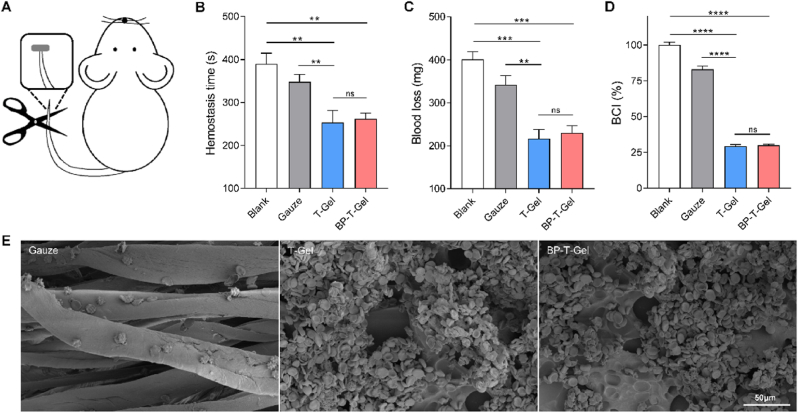
Hemostatic capacity of BP-T-Gel. (A) Schematic illustration of hemostatic model by amputating mice tail. Hemostatic time (B) and blood loss (C) of model mice treated by gauze, T-Gel and BP-T-Gel, respectively. Blank, no treatment. (D) Whole blood clotting evaluation. (E) SEM images of hemocyte adhesion on gauze, T-Gel and BP-T-Gel. Data are presented as means ± SD (n = 3). ∗∗*p* < 0.01, ∗∗∗*p* < 0.001, ∗∗∗∗*p* < 0.0001. ns, no significant difference.

Considering the critical importance of hemostasis in evaluating wound dressing materials, as excessive blood loss from trauma can be life-threatening, we utilized a mouse hemorrhaging tail model to assess the hemostatic performance of the hydrogel (Fig. 7A). Compared to the untreated control group (blank) or the gauze-treated group, applying hydrogel pieces significantly reduced both hemostatic time and blood loss (Fig. 7B and C). Notably, there were no significant differences in hemostatic time and blood loss between T-Gel and BP-T-Gel treatments, suggesting that BP has a minimal impact on the hemostatic performance of the hydrogel. To explore the hemostasis mechanism further, we measured the blood clotting ability by dropping red cell suspension on different samples and incubating them for 3 min at 37 °C. As shown in Fig. 7D, the blood-clotting index (BCI) of the hydrogel was approximately 30 %, significantly lower than that of gauze (85 %). This lower index indicates a more effective blood-clotting capacity. Additionally, SEM observations revealed that a large number of red blood cells aggregated on the surface of the hydrogel, while only a few adhered to the gauze (Fig. 7E). These findings suggest that the hydrogel holds great potential as an effective hemostatic material for wound dressings.

### 3.8 Wound disinfection and healing tests

**Fig. 8:**
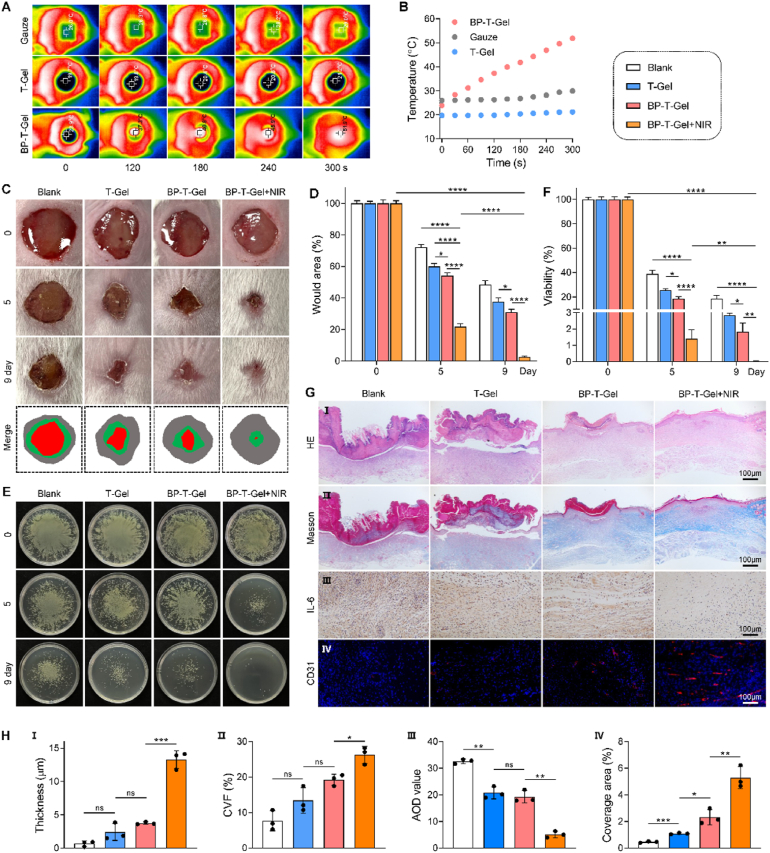
Therapeutic effect of BP-T-Gel in CRPA-accompanied wound infection. Thermal infrared images (A) and relative temperature curves (B) of mice treated with Gauze, T-Gel and BP-T-Gel under 808 nm NIR irradiation. Representative skin wound photographs (C) and wound closure rates (D) on indicated time. Represent photographs of agar plates (E) and colonies counts (F) of CRPA bacteria in wound tissues from mice treated with different samples. (G) Representative bright-field images of H&E staining (I), Masson's trichrome staining (II), immunohistochemistry staining of IL-6 (III), and immunofluorescence images of neovascularization in wound tissues after being stained of CD 31 with red color (IV). (H) Quantitative analysis of epidermal thickness (I), collagen volume fraction (CVF) (II), IL-6 positive cell (III), and the relative coverage area of CD31 (IV). ns, no significant difference. Data are presented as mean ± SD (n = 3). ∗*p* < 0.01, ∗∗*p* < 0.01, ∗∗∗*p* < 0.001, ∗∗∗∗*p* < 0.0001. ns, no significant difference.

Inspired by their excellent mechanical properties and remarkable biological activities, we further explored the *in vivo* applicability of the hydrogel using a dorsal-skin incised mouse model with wounds infected by CRPA. As shown in Fig. 8A and B, thermal imaging revealed that after 4 min of NIR irradiation, the temperature in the wound area treated with BP-T-Gel increased to 45.9 °C. To verify the safety of this temperature for normal skin tissue, H&E and Masson staining were performed on skin tissues obtained from healthy mice after treated with BP-T-Gel + NIR. As shown in Fig. S13A, the staining patterns of the BP-T-Gel + NIR-treated group were indistinguishable from those of the healthy control group. The skin structures were well-preserved, with no significant cellular damage evident. Additionally, numerous studies have extensively reported that this temperature (45.9 °C) does not cause harm to the animal and is considered an adaptive photothermal temperature [[53], [54], [55]]. This precise local hyperthermia minimized potential harm to surrounding healthy tissues. Based on these findings, we decided to use a 4-min irradiation duration at this power level for the subsequent treatments. After 24 h of infection (day 0), redness and swelling appeared around the wounds in all groups, along with some degree of pus formation (Fig. 8C). Compared to the blank control group, the wounds in the other three groups were noticeably smaller by day 5. Notably, the wound size in the BP-T-Gel + NIR group significantly decreased at various time points post-treatment, with nearly no open wounds observed after 9 days. The calculation of wound size confirmed that BP-T-Gel + NIR provided the best wound healing efficacy among all treatments (Fig. 8D). To evaluate the antibacterial activity of the hydrogel, skin tissue samples from the wounds were collected, and viable bacteria were estimated using the plate counting method. As illustrated in Fig. 8E and F, the BP-T-Gel + NIR group showed almost no bacterial colonies, demonstrating its superior therapeutic effect. H&E staining of main organs (heart, liver, spleen, lung, and kidney) in Fig. S13B showed no obvious organ damage or inflammatory lesions, demonstrating that the advanced hydrogel not only efficiently promotes the wound healing but also features good *in vivo* biosafety.

Histological examination was conducted to evaluate the wound healing process. Wound tissues from different treatment groups were stained with hematoxylin and eosin (H&E) on day 9 after therapy (Fig. 8G–I and 8H-I). Compared to other groups, the BP-T-Gel + NIR group exhibited more regenerated dermal tissues, including new skin appendages such as dermal fibroblasts, and the thickness of the regenerated epithelial tissue in the BP-T-Gel + NIR group was the greatest, indicating enhanced re-epithelialization. To further assess the healing process, Masson's trichrome staining was used to evaluate collagen and granulation tissue formation. Collagen fibers and granulation tissues, which both contribute to faster wound healing, were stained blue and red respectively. As shown in Fig. 8G–II and 8H-II, significant collagen and granulation tissue deposition was observed in the T-Gel and BP-T-Gel groups, suggesting that the wounds were progressing well towards recovery. Notably, the BP-T-Gel + NIR group displayed more uniform collagen bundles and dermis. Quantitative image analysis showed that the collagen volume fraction (CVF) in the BP-T-Gel + NIR group was significantly higher than that in the other three groups, suggesting that BP-T-Gel + NIR treatment significantly enhances extracellular matrix remodeling and tissue reconstruction. Furthermore, immunohistochemical staining of the inflammatory cytokine IL-6 showed that BP-T-Gel reduced inflammation at the wound site, with significantly lower IL-6 level compared to the control group (Fig. 8G–III and 8H-III), suggesting that the BP-T-Gel exhibited strong anti-inflammatory properties. Angiogenesis is also a key step in wound healing and tissue remodeling, with CD31 being a common marker for blood vessel formation [56]. Immunofluorescence staining revealed a significant increase in CD31 expression at the wound site in the BP-T-Gel group (Fig. 8G–IV and 8H-IV), which can be attributed to the enhanced and accelerated wound repair process promoting angiogenesis. These results collectively demonstrated the superior ability of BP-T-Gel to promote wound healing *in vivo*.

## 4 Conclusion

To summarize, we have successfully developed a multifunctional dextran-based hydrogel platform that efficiently captures and eliminates CRPA on wounds. By grafting galactose and fucose (glycohooks) onto the 3D hydrogel and 2D BP nanosheets, we significantly enhance the selective capture of CRPA through multivalent carbohydrate-lectin interactions. Additionally, these glycoligands improve the stability of BP nanosheets, making them less prone to degradation. Thanks to these glycohooks, the BP-T-Gel can spontaneously catch bacteria, localizing thermal ablation at the site of infection. This not only enhances bactericidal performance but also minimizes side effects on nearby healthy tissue. The inclusion of magnolol and boronate esters in the hydrogel structure provides sustained antioxidant capabilities. Due to multiple dynamic bonds, the hydrogels exhibit promising self-healing properties, good mechanical strength, and moderate tissue adhesion. Moreover, the hydrogels demonstrate multifunctional bioactivities, including excellent biocompatibility and hemostasis. All these advantages make BP-T-Gel a promising solution for managing wound infections. In this study, we use glycohook and glycohook-equipped hydrogels to strengthen the photothermal effects of BP nanosheets, offering a design strategy that can be applied to precisely treat other infections by enhancing PTT of various nanomaterials.

## CRediT authorship contribution statement

**Peng Jiang:** Writing – original draft, Methodology, Data curation. **Shuoyi Zhang:** Methodology, Investigation. **Zheng Tang:** Software. **Weilan Wang:** Formal analysis. **Haibo Mu:** Writing – review & editing. **Kaixu Chen:** Writing – review & editing, Supervision, Project administration, Funding acquisition, Conceptualization.

## Declaration of competing interest

The authors declare the following financial interests/personal relationships which may be considered as potential competing interestsKaixu Chen reports financial support was provided by Xinjiang Uygur Autonomous Region Department of Science and Technology. If there are other authors, they declare that they have no known competing financial interests or personal relationships that could have appeared to influence the work reported in this paper.

## Data Availability

Data will be made available on request.
