# Supplementary material for: Glyco-engineered multifunctional hydrogel with bacterial fishing hooks for targeted bacterial eradication and enhanced wound healing
Source: Mater Today Bio. 2025 Aug 5;34:102174. doi: 10.1016/j.mtbio.2025.102174 (PMC12345368; doi:10.1016/j.mtbio.2025.102174)
Supplement: Multimedia component 1 [file mmc1.docx]

Supplementary material

**Glyco-engineered multifunctional hydrogel with bacterial fishing hooks for targeted bacterial eradication and enhanced wound healing**

Peng Jiang^1#^, Shuoyi Zhang^1#^, Zheng Tang^1^, Weilan Wang^2^, Haibo Mu^3^, Kaixu Chen^1^*

^1^ Xinjiang Key Laboratory of Herbivore Nutrition for Meat&Milk, College of Animal Science, Xinjiang Agricultural University, Urumqi 830052, China

^2^ Xinjiang Key Laboratory of Biological Resources and Genetic Engineering, College of Life Science and Technology, Xinjiang University, Urumqi 830046, China

^3^ College of Chemistry & Pharmacy, Northwest A&F University, Yangling 712100, China

^#^ These authors contributed equally to this work

* Correspondence

Kaixu Chen, PhD

E-mail: chenkaixu@xjau.edu.cn

**1. Methods**

**1.1. Chemical synthesis of Gal-NH_2_ and Fuc-NH_2_**

General materials: ACS-grade reagents and chemicals were obtained from Aladdin, Aldrich, or Tansoole, and used without further purification unless otherwise stated. When necessary, acetonitrile was bought anhydrous and stored over molecular sieves (4 Å).

Synthesis of Gal-NH_2_ and Fuc-NH_2_: As shown in scheme 1, to prepare amino galactose (Gal-NH_2_, **8**) and amino fucose (Fuc-NH_2_, **9)**, we developed the synthetic route by adapting the synthetic procedures previously described[1, 2].


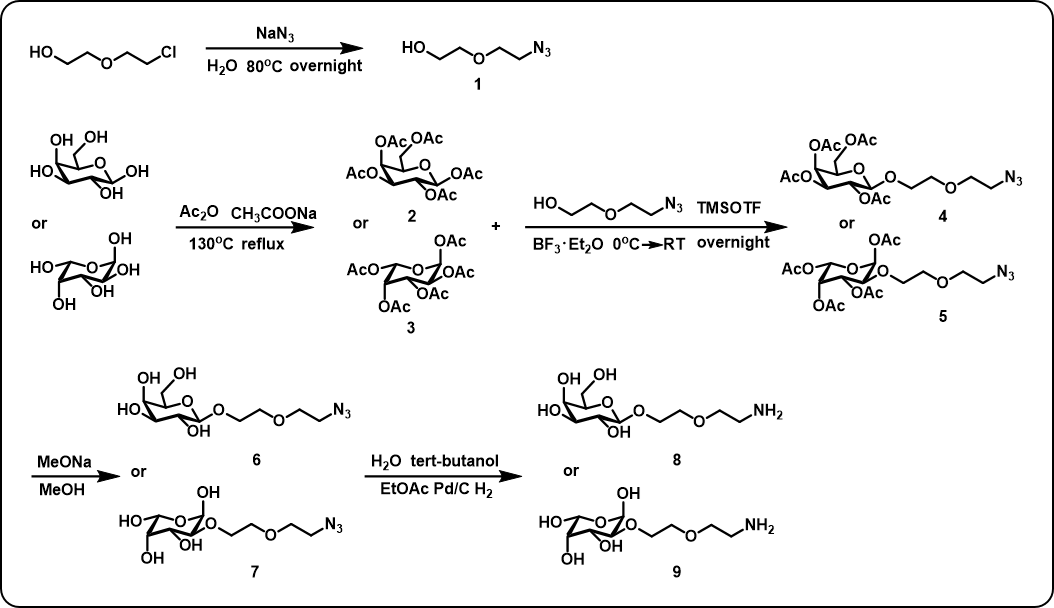


**Scheme S1**. Synthesis of Gal-NH_2_ (**8**) and Fuc-NH_2_ (**9)**.

Compound **1**. 2-(2-chloroethoxy)ethan-1-ol (1 mL, 9.47 mmol) was dissolved in H_2_O (10 mL) and sodium azide (1.5 g, 23.7 mmol, 2.5 eq) was added. The reaction mixture was stirred at 80 ºC under N_2_ overnight, and then poured into sodium hydroxide (5%, 30 mL) and extracted with diethyl ether (30 mL 🞨 3). The organic layer was dried over Na_2_SO_4_ and evaporated to dryness to afford **1** (1 g, 80%) as a colorless oil.

Compound **2** and **3**. To a solution Sodium acetate (1 g, 12.2 mmol) in Acetic anhydride (150 mL) was added D-Galactose (2.34 g, 13 mmol) at RT. After being heated under reflux for 1.5 h, the reaction solution was cooled to RT slowly and added CH_2_Cl_2_ (100 mL). The mix was washed successively with water (3 🞨 100 mL), NaHCO_3_ (3 🞨 100 mL), and brine (3 🞨 100 mL). The organic phase was then dried (Na_2_SO_4_), filtered and concentrated in vacuo to afford compound **2** as a yellow oil. And the compound **3** was synthesized in the same way as compound **2** using fucose as the reactant.

Compound **4** and **5**. To an ice-cold (0 °C) solution of D-Galactose peracetate **2** (844.29 mg, 2.16 mmol, 1 eq) and **1** (567 mg, 4.3 mmol, 2 eq) in dry acetonitrile (30 mL) under N_2_ atmosphere was added dropwise boron trifluoride diethyl etherate (528 μL, 4.3 mmol, 2 eq) and trimethylsilyl trifluoromethanesulfonate (80 μL, 0.43 mmol, 0.2 eq). The reaction mixture was allowed to warm to room temperature and then stirred overnight under N_2_. The solution was quenched with a saturated solution of NaHCO_3_ (30 mL) and diethyl ether (30 mL). The organic phase was then washed with NaHCO_3_ (2 🞨 30 mL), H_2_O (30 mL) and brine (30 mL). It was dried over Na_2_SO_4_, filtered and concentrated under reduced pressure. Purification of the residue by flash chromatography on silica gel (PE/EA 10:1-5:1) afforded the desired mannoside 3 as a yellow oil. And the compound **5** was synthesized in the same way as compound **4** with the compound **3** as the reactant.

Compound **6** and **7**. To a solution of **4** (500 mg, 1.09 mmol, 1 eq) in dry MeOH (20 mL) under N_2_ atmosphere was added sodium methoxide (234 mg, 4.34 mmol, 4 eq) at room temperature and the solution was stirred during 2 h. The reaction mixture was concentrated under reduced pressure. Purification of the residue by flash chromatography on silica gel (DCM:MeOH 6:1) afforded the desired mannoside **6** as a yellow oil. And the compound **7** was synthesized in the same way as compound **6** with the compound **5** as the reactant.

Compound **8** and **9**. Add a catalytic amount of Pd/C (5.321 mg) catalyst to a stirred solution of **6** (293.28mg, 1 mmol, 1eq) in water (20 mL), t-Butanol (20 mL) and EtOAc (1 mL) under H2 atmosphere. And then stir the reaction overnight. At last filter the reaction mixture. The fractions containing the product were concentrated as a yellowish oil (216.5 mg, 81%). And the compound **9** (197.54 mg, 78%) was synthesized in the same way as compound **8** with the compound **7** as the reactant.

NMR spectra were recorded on a Bruker 500 MHz Spectrometer with working frequencies of 500 MHz for ^1^H, respectively, in CDCl_3_ or CD_3_OD. The residual signals from CD_3_OD (^1^H: δ 3.31 ppm) were used as internal standards. And all of the nuclear magnetic spectrums were resolved with MestReNova software. Chemical shifts (δ) were referred to the partially deuterated nuclei of the solvents used. All spectra were described in the first order. The chemical shifts of signals featuring defined multiplicity were determined by the arithmetic mean of the signal lines. The following abbreviations were used: s = singlet, d = doublet, t = triplet, q = quartet, m = multiplet and their combinations.


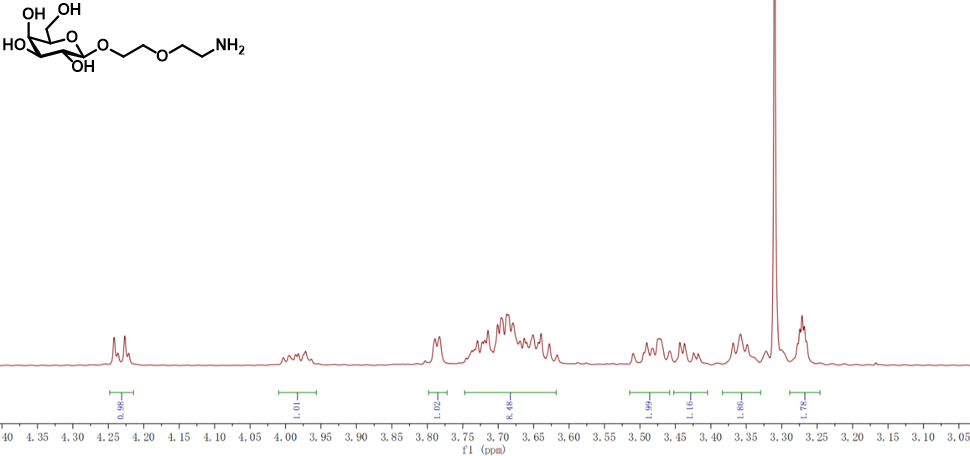


**Fig. S1**. ^1^H NMR spectrum of Compound **8** in MeOD. ^1^H NMR (500 MHz, Methanol-d4), δ (ppm): δ 4.23 (dd, J = 7.6, 2.8 Hz, 1H), 4.01 – 3.96 (m, 1H), 3.79 (dd, J = 3.4, 1.1 Hz, 1H), 3.73 (ddd, J = 7.5, 4.9, 3.1 Hz, 1H), 3.71 – 3.61 (m, 4H), 3.51 – 3.45 (m, 2H), 3.43 (ddd, J = 9.9, 3.5, 1.1 Hz, 1H), 3.36 (t, J = 5.0 Hz, 2H), 3.27 (p, J = 1.7 Hz, 2H).


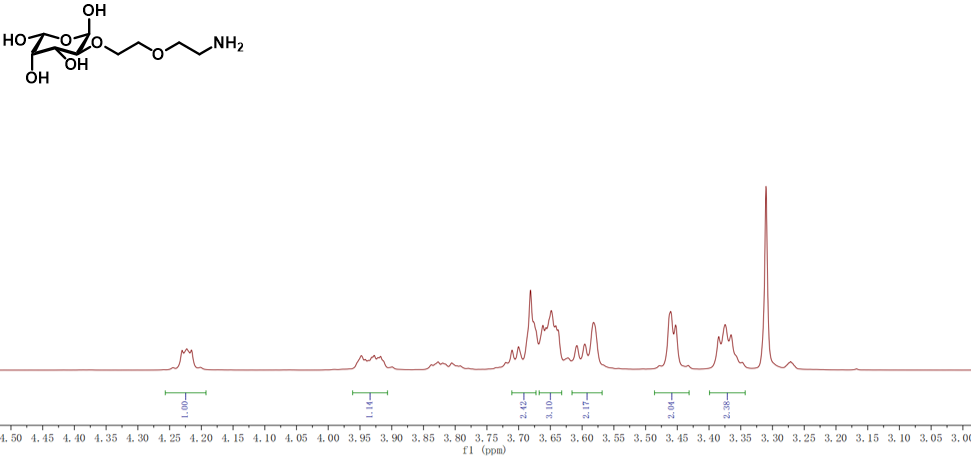


**Fig. S2**. ^1^H NMR spectrum of Compound **9** in MeOD.^1^H NMR (500 MHz, Methanol-d4), δ (ppm): δ 4.26 – 4.19 (m, 1H), 3.93 (ddt, J = 12.1, 4.5, 1.9 Hz, 1H), 3.71 – 3.67 (m, 2H), 3.65 (td, J = 5.1, 2.2 Hz, 3H), 3.62 – 3.57 (m, 2H), 3.49 – 3.43 (m, 2H), 3.37 (t, J = 5.0 Hz, 2H).

**1.2. Characterization of glyco@BP**

The size distribution of BP nanosheets and glyco@BP nanosheets in the aqueous solution (50 μg/mL) was studied by dynamic light scattering (DLS, ZEN3600, Malvern Instruments Limited, Malvern, England). The morphology was analyzed by transmission electron microscopy (TEM, FEI Talos F200X, Hillsboro, USA). Atomic force microscope measurement was performed on a Bruker Dimension Icon (AFM, Bruker, Germany). The elemental compositions and chemical status of glyco@BP nanosheets were analyzed by X-ray photoelectron spectroscopy (ThermoFisher Scientific, ESCALAB 250Xi, Waltham, USA). UV-vis-NIR spectrum was measured by a spectrometer (HITACHI, 3900H, Hitachi, Japan).

**1.3. Characterization of the hydrogels**

The Fourier transform infrared spectra (FTIR) were recorded with a spectrophotometer (ThermoFisher, Nicolet 6700, Waltham, America) equipped with an attenuated total reflection (ATR) accessory. For scanning electron microscopy (SEM, Nano SEM-450, Hillsboro, America) observation, the freeze-dried hydrogel blocks were fractured through liquid nitrogen freezing to observe cross-section. The surfaces of freeze-dried hydrogel films were observed directly. All the samples were treated by spray-gold before observation. The chemical components of BP T-Gel were analyzed by EDS. The EDS data were obtained using SUTW-Sapphire detector (EDAX, Mahwah, USA) with a voltage of 200 kV and a resolution of 136.65.

**1.4. Rheological tests**

Rheological measurement was performed using a rheometer (DHR-1, Waters, Milford, America) with a parallel-plate geometry (10 mm in diameter, 2 mm between gap distance). The frequency sweep was taken at a constant strain of 0.1% by a frequency varying from 0.1 to 100 Hz at 25°C. The strain sweep was taken at a constant frequency of 1 Hz by a strain varying from 0.1% to 100% at 25 °C and 60 °C.

**1.5. Swelling ratio and porosity of hydrogels**

The swelling ratio of hydrogels were measured according to the reported gravimetric method [3]. Briefly, different hydrogels of equal weights (Wi) were immersed in deionized water at 37 °C. At regular intervals, hydrogels were taken out and immediately weighed (W_t_) after gently blotting the surface water with filter paper. The swelling ratio was calculated using the following equation: Swelling ratio = (W_t_ -W_i_)/W_i_ × 100%.

The porosity of hydrogels was measured by liquid displacement. Ethanol was selected as the displacement liquid as it permeates through the scaffolds without swelling or shrinking the matrix. The scaffolds (dry weight, W_d_) were immersed in the ethanol under vacuum for 5 min and the weights of scaffolds in ethanol were recorded as W_l_. The scaffolds were taken out and the liquid on the surface was removed by filter paper. The weight of the wet scaffold was recorded as W_w_. The porosity was be obtained by: Porosity (%) = (W_w_ – W_d_)/ (W_w_- W_l_) × 100%

**1.6. Mechanical tests**

All the tensile tests and loading-unloading tests were performed using an electronic universal tester (MTS, CMT6103, Eden Prairie, USA). For the tensile test, the cylindrical samples (20 mm in length and 10 mm in diameter) were tested with 10 mm initial distance between two clamps, and the strain speed was constant at 100 mm/min. For the loading-unloading tests, the cylindrical samples (20 mm in length and 10 mm in diameter) were tested with 10 mm initial distance between two clamps and the strain speed was constant at 100 mm/min. The samples were initially stretched to a predetermined strain (1000%) and then unloaded at the same velocity (100 mm/min). The compression properties of samples were tested using the cylindrical samples (20 mm in length and 10 mm in diameter) were tested with 10 mm initial distance between two clamps, and the strain speed was constant at 1 mm/min. All the mechanical tests were performed at 25 °C in air.

**1.7. Self-healing and adhesive properties of BP T-Gel**

To evaluate the self-healing capability, the hydrogels (20 mm in length and 10 mm in diameter) was cut into two pieces, and the separate parts were brought into contact in a PET mold and placed at room temperature for 10 min. To evaluate the healing efficiency, tensile strengths of the original and self-healed hydrogels were investigated as above. To evaluate adhesive properties, BP T-Gel was cut into rectangular pieces with a dimension of 20 mm ×10 mm × 2 mm and applied to skin, glass and metal respectively. After 5 min, using spring dynamometer was conducted manually to test the adhesive strength of the hydrogels on different surfaces. For Fig. 2E and S6, author Peng Jiang was the subject of experiments, and an informed consent was obtained. All experimental were conducted in accordance with the Code of Ethics of the World Responses to Technical Check Results Medical Association (Declaration of Helsinki) for experiments involving humans, and were approved by the Ethics Review Executive Committee of Northwest A&F University.

**1.8. Stability evaluation of glyco@BP nanosheets**

The UV-Vis absorption spectra and photothermal curves of BP and glyco@BP nanosheets in the presence of ambient water was recorded to assess their stability for 20 days. The morphological changes were observed using TEM to analyze the oxidation state of bare BP and glyco@BP nanosheets after storage for 20 days.

**1.9. Live/Dead staining assay**

The live bacteria were stained green by SYTO 9 and the dead were stained red by PI. In brief, 100 μL of bacteria (0.4 OD_600_) in PBS was incubated with Gel or BP T-Gel under 808 nm NIR laser for 15 min (1W/cm^2^). Then 900 μL of PBS was added to rinse the bacteria adhered to the surface of the hydrogels. Afterward, propidium iodide (PI, 10 μL, 10 μg/mL) and SYTO 9 (10 μL, 10 μg/mL) fluorescent dyes were introduced to 200 μL of bacteria solution and cultured in the dark for 15 min. All samples were observed under an inverted fluorescence microscope.

**1.10. Morphological characterization of bacteria**

The morphological changes of the bacteria were visualized by SEM. After treatment as antibacterial assay, the hydrogel-bacteria conjugate samples were washed with PBS, fixed overnight with glutaraldehyde (2.5%), and dehydrated by sequential treatment of ethanol solutions (50, 60,70, 80, 90 and 100%) for 10 min, respectively. Before SEM observation, the samples were sputter-coated with platinum.

**1.11. Antioxidant activity**

The antioxidant efficiency of hydrogels was evaluated by DPPH free radical scavenging assay and intracellular reactive oxygen species scavenging assay [4].

For DPPH assay, the hydrogels were ground by using a tissue grinder. Afterward, 100 µM DPPH and different amounts of the hydrogel samples (10 - 100 mg) were dispersed in 2 mL ethanol. The mixture was stirred and incubated in a dark place at 37 °C for half an hour. The optical absorption at 517 nm were recorded and the degradation of DPPH was calculated by the following formula:

DPPH scavenging = (A_Blank_ - A_Hydrogel_) /A_Blank_ ×100%

where A_Blank_ and A_Hydrogel_ are the absorption of the Blank (DPPH + ethanol) and the

absorption of the Hydrogel (DPPH + ethanol + hydrogel), respectively.

For intracellular ROS assay [5], L929 cells (3 × 10^5^ cells per well) were seeded with hydrogel granule in 6-well plates overnight and then supplemented with 100 μM of H_2_O_2_. After incubation for 2 h, the medium was removed and the plate was washed with PBS, 500 μL medium containing 10 mM DCFH-DA (Solarbio, Beijing, China) was added to each well and incubated for 20 min. The Intracellular ROS content was measured using a fluorescence microscope (DMi8, Leica, Wetzlar, Germany).

**1.12. Cytotoxic tests**

The hydrogel extracts were prepared as previous reported [3]. In general, the prepared cylindrical hydrogels (30 mm in length and 10 mm in diameter) were soaked in PBS at 37 °C for 24 h to achieve full swelling and then sterilized by autoclaving. After that, 1 g of the sterilized hydrogel was immersed in 5 mL DMEM medium at 37 °C for 24 h. The hydrogel extracts were collected after removing the hydrogel.

Cell viability was evaluated against L929 cells by MTT assay. Cells (5.0×10^3^ per well) were planted in a 96-well plate and incubated overnight. Then, the medium was replaced by the hydrogel extracts medium and incubated for different times (12 - 36 h). PBS was used as blank control (100% viability) and phenol as positive control. Cell viability was quantified using MTT as previous [3]. For live/dead staining, L929 cells were pretreated with hydrogel extracts as above for 24 h, then stained using Annexin V-FITC/PI kit, and observed by a DMi8 fluorescence microscope.

The dispersion of BP or glyco@BP (100 µg/mL) from different degradation times was centrifuged at different time points, and the supernatant was collected and sterilized by filtration. The cytotoxicity of the degradation products was determined as above by adding 10% the sterilized supernatant to the cell culture medium.

**1.13. *In vitro* hemolysis assay**

Hemolysis assay was conducted according to a previous method [6]. In brief, red blood cells (RBCs) were separated from fresh mouse blood, washed with normal saline three times via centrifugation (8000 rpm for 5 min), Subsequently, the RBCs were diluted tenfold with equivalent saline to prepare the RBCs dispersion. After that, 1 g of the Gel and BP T-Gel was immersed in 5 mL normal saline for 24 h, respectively. The hydrogel extracts were obtained after removing the hydrogel to prepare the hydrogel dispersion. Then the RBCs dispersion (0.2 mL) was gently mixed with Gel and BP T-Gel dispersions (0.8 mL) respectively. The RBCs incubated with deionized water acted as a positive control, and with saline as a negative control. All the samples were incubated at 37 °C for 4 h, and then centrifugated at 8000 rpm for 5 min, the absorbance of supernatant was measured at 492 nm by a multifunctional microplate reader. the hemolysis ratio was calculated by the following equation:

Hemolysis ratio = (A_S_-A_NC_/A_PC_-A_NC_) ×100%

where A_S_, A_NC_, and A_PC_ represented the absorbance values of the sample, the negative control, and the positive control, respectively.

**1.14. Cell migration experiment**

Cell migration was investigated by incubating L929 cells with hydrogel extracts as reported [7]. Initial cell seeding density was 2.0×10^5^ cells per well. Subsequently, a linear wound was made by scratching using a cell scraper. Then, cells were rinsed with DMEM medium and incubated with hydrogel extracts for another 12 or 24 h. PBS was used as blank control. Cells were visualized by Leica DMi8 microscope. The migration area was calculated using following equation:

Migration area = (A_t_ - A_0_) / A_0_ ×100%

where A_0_ is the blank control of blank area at 0 h and A_t_ is the experimental groups at indicated time.

**1.15. M2 polarization of macrophages**

RAW 264.7 macrophages were cultured in DMEM supplemented with hydrogel extract for 24 hours, with lipopolysaccharide (LPS) serving as the control group. After cultivation, RAW 264.7 cells were collected and stained with F4/80, CD86, and CD206 antibodies, and analyzed using flow cytometry. The levels of interleukin-6 (IL-6) and interleukin-10 (IL-10) in the culture supernatants were measured using ELISA kits [8].

**1.16. Histological analysis**

The wound tissue was fixed in 4% paraformaldehyde for 48 hours, after which the tissue was embedded in paraffin and sectioned into 5-μm thick slices. Hematoxylin and eosin (H&E) staining was performed to evaluate the width of the granulation tissue, while immunohistochemistry for interleukin-6 (IL-6) was used to assess the level of wound inflammation. Additionally, Masson’s trichrome staining was employed to quantify collagen deposition. For immunofluorescence analysis, the tissue sections were first de-paraffinized, followed by antigen retrieval and blocking. The sections were then incubated with primary antibodies, specifically rabbit monoclonal anti-CD31. Subsequently, the sections were incubated with the appropriate secondary antibodies, and the nuclei were stained with DAPI. Finally, ImageJ software was used to quantify the staining results [9].

**1.17. Degradation behaviour of hydrogel**

As reported [10], to evaluate the *in vitro* degradability, 1 g of freeze-dried hydrogel (W_0_) was immersed in 20 mL of PBS at 37 °C, then periodically retrieved, rinsed with ionized water and promptly freeze-dried. The remaining mass was recorded as W_t_. The remaining mass percentage was used to indicate the degradation rate: Remaining Mass (Weight %) = W_t_ / W_0_ × 100.

**1.16. Degradation of glyco@BP**

The total phosphorus content was determined using the ammonium molybdate spectrophotometric method [11]. First, 1 mL of a 100 µg/mL BP or glyco@BP solution was centrifugally dispersed and sampled at different time intervals. A 50 mg/mL potassium persulfate solution was added, followed by high-pressure digestion at 121 °C for 30 min. After cooling, the volume was adjusted, and 1 mL of the digested solution was taken. Ammonium molybdate-sulfuric acid reagent (5 M H_2_SO_4_) and ascorbic acid (100 mg/mL) were added sequentially. After 15 min of color development, absorbance was measured at 700 nm. Phosphate concentration was quantified using a standard curve (0-30 μg/mL).

**1.17. Calcification in L929 cells**

The Cresol Red S assay kit was used to detect calcification in L929 cells [12]. First, L929 cells were plated into a 96-well plate and cultured until they reach an appropriate density. Subsequently, the degradation supernatants of Glyco@BP were added. Next, an Alizarin Red S dye solution was introduced, and the cells were incubated for 60 min to enable the dye to bind to the calcified deposits. After that, the excess dye was removed by washing with PBS, and a dissolving solution was added to solubilize the deposited dye. Finally, the absorbance of the solubilized solution was measured at 560 nm using a microplate reader, and the degree of calcification was determined.


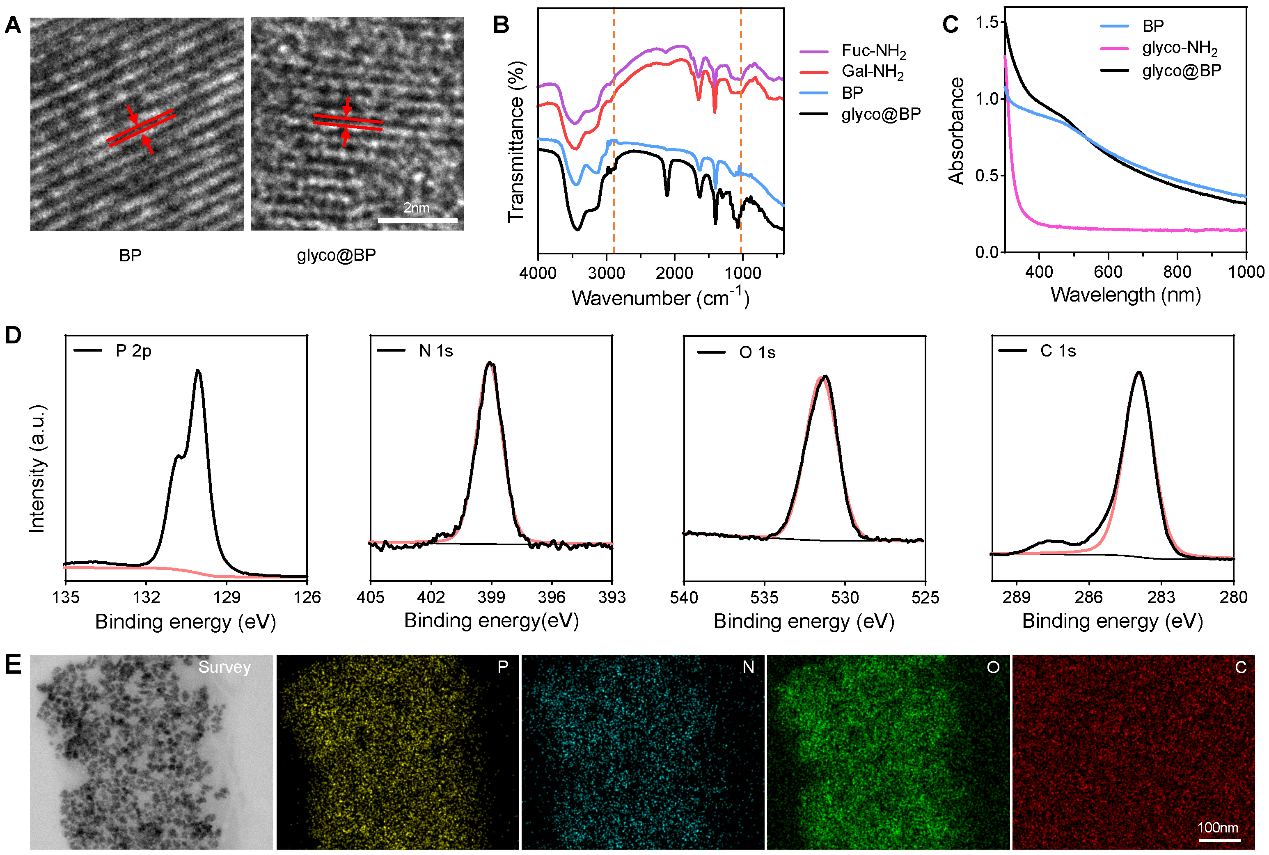


**Fig. S3.** (A) HR-TEM image of BP nanosheets and glyco@BP. (B) FT-IR spectra of the as-prepared Gal-NH_2_, Fuc-NH_2_, BP nanosheets and glyco@BP. (C) UV-vis-NIR absorption spectrum of BP nanosheets and glyco@BP. (D) XPS spectra of glyco@BP: all elements and high-revolution spectra of C 1s, N 1s, O 1s and P 2p. (E) STEM-EDX images of glyco@BP.


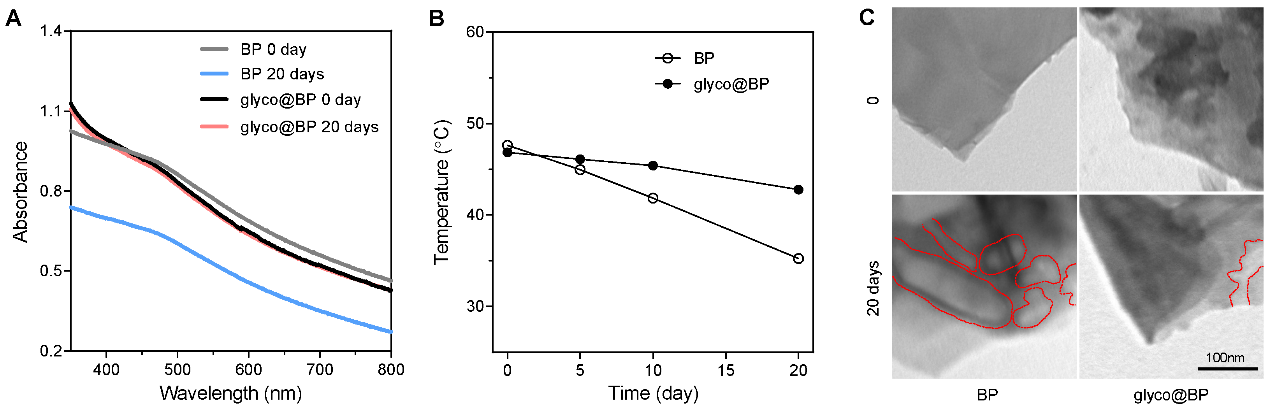


**Fig. S4.** (A) UV-vis-NIR absorption spectra of BP nanosheets and glyco@BP after exposure to water for 20 days. (B) Photothermal heating curves of bare BP nanosheets and glyco@BP for 30min after dispersed in water for 0, 5, 10, 20 and 30 days. (C) The TEM images of BP nanosheets and glyco@BP in water for 20 days. Scale bar, 100 nm.


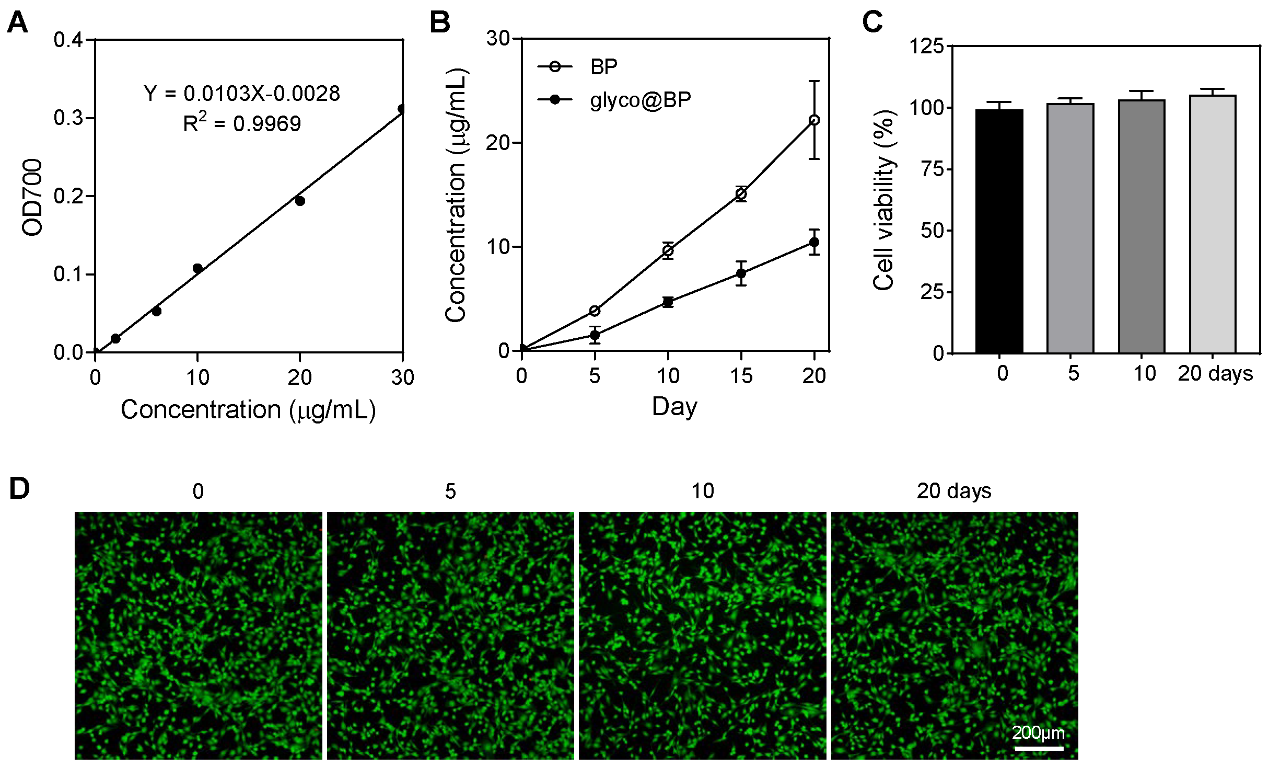


**Fig. S5.** The total phosphorus content in the solution was determined using the ammonium molybdate spectrophotometric method. (A) The total phosphorus standard curve. (B) Total phosphorus content in the centrifuged supernatant of BP and glyco@BP on different days. Biocompatibility and biological properties of the degradation products of glyco@BP. Cell viability (C) and Live/dead staining (D) and of L929 cells treated with degradations from different durations.


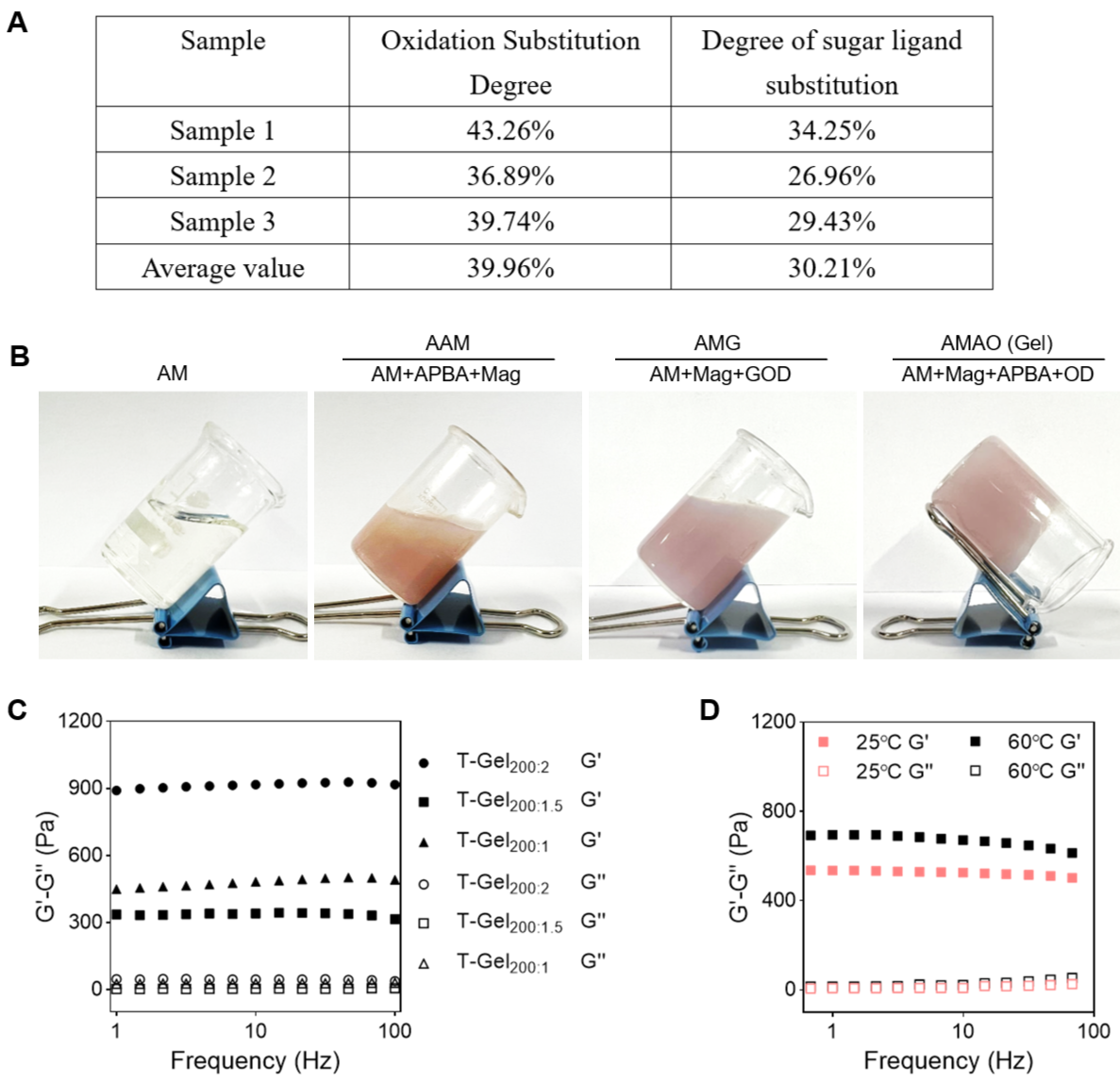


**Fig. S6.** (A) Oxidation substitution degree of oxidized dextran and substitution degree of sugar ligand in GOD. (B) The different ingredients’ photographs of the resultant solution. (C) Rheology properties of T-Gel with different contents of Magnolol, APBA, GOD with oscillatory frequency sweep by 0.1% strain. (D) Rheology properties of T-Gel with oscillatory frequency sweep by 0.1% strain at 25 °C and 60 °C.


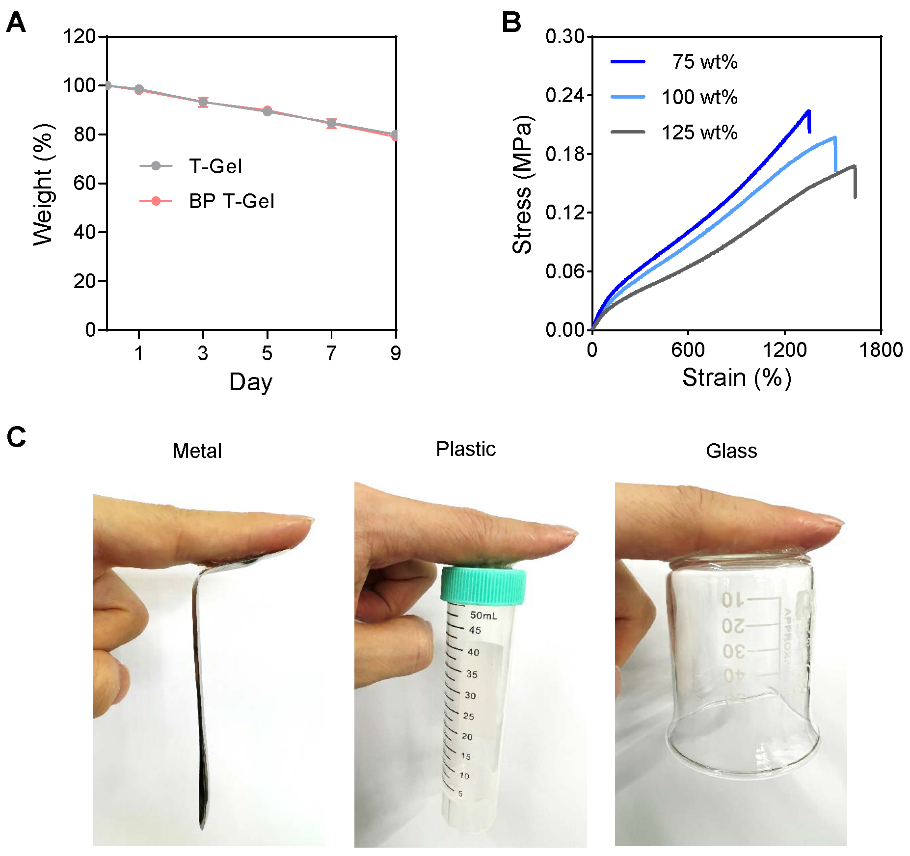


**Fig. S7.** (A) The degradation behaviour of T-Gel and BP-T-Gel after soaking in water for different times. (B) The tensile strain curves of BP-T-Gel after swelling in water at 75, 100, and 125 wt%. (C) BP-T-Gel glued metal, plastic and glass to the author’s skin.


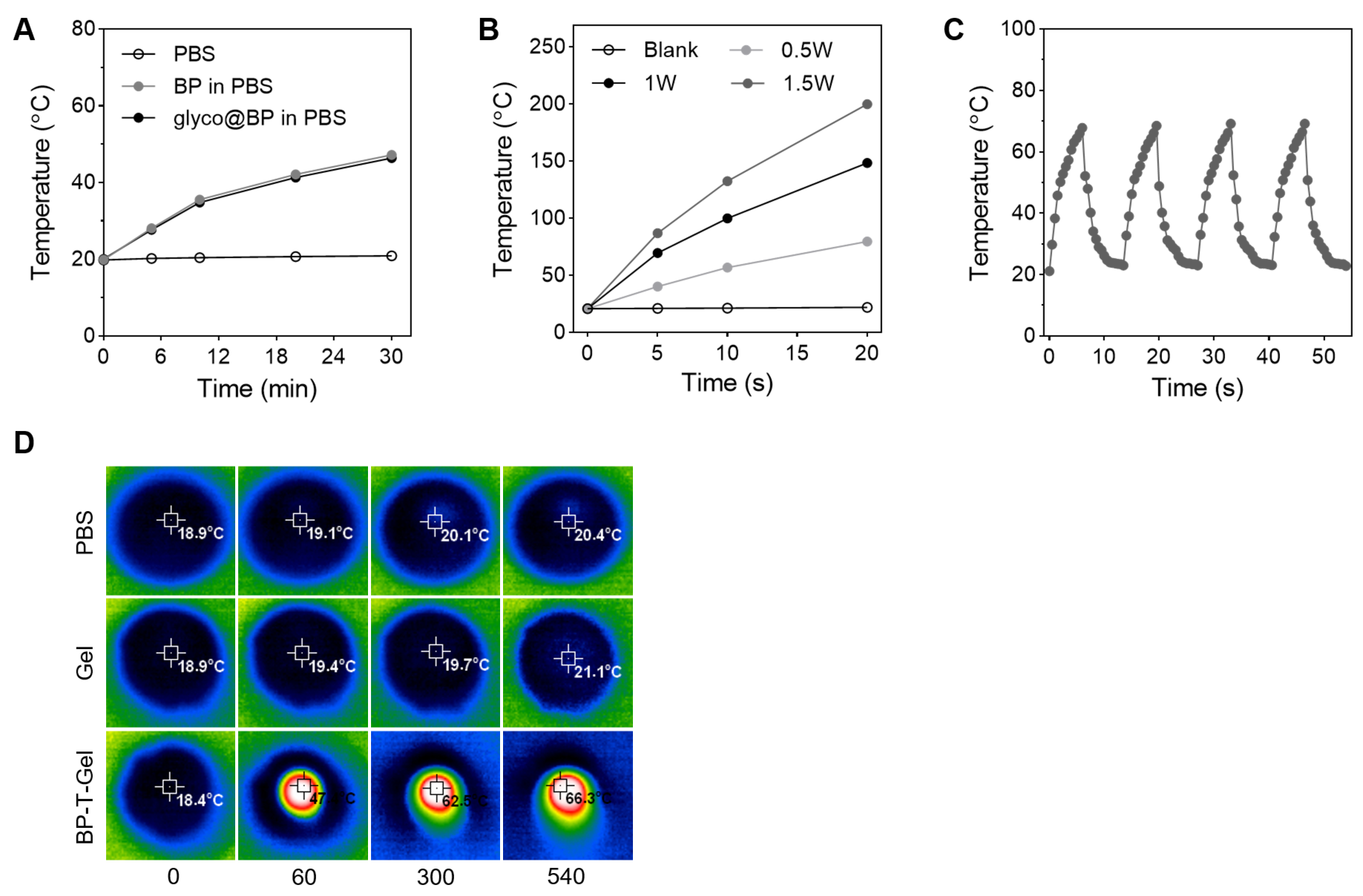


**Fig. S8.** (A) Temperature curves of BP nanosheets and glyco@BP in PBS under 808 nm NIR irradiation. NIR power 1 W/cm^2^. (B) The corresponding time-dependent temperature curves of glyco@BP powder with NIR irradiation of different power. (C) Transient temperature of glyco@BP powder under repeated irradiation on-off of 808 nm NIR irradiation (1 W/cm^2^). (D) Thermal infrared images of BP-T-Gel under NIR irradiation with a power of 1W/cm^2^.


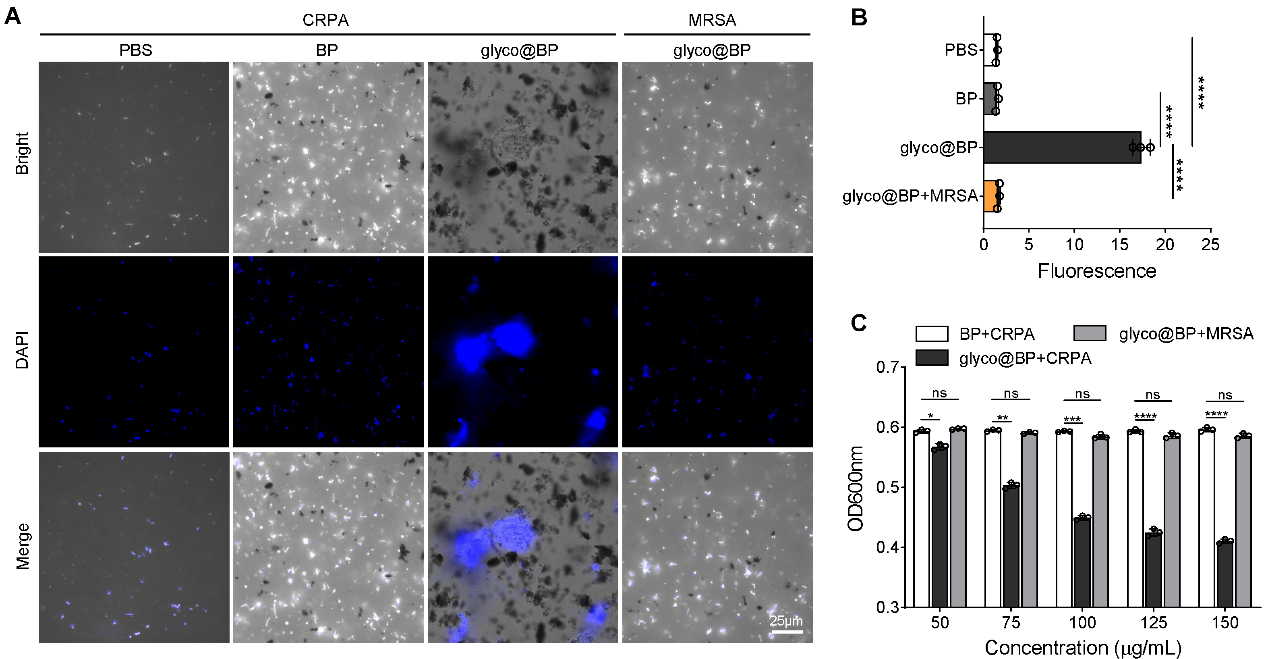


**Fig. S9.** Fluorescence images (A) and relative fluorescence intensity (B) of bacteria after treatment with BP or glyco@BP. DAPI (blue) was used to stain bacteria. (C) OD_600_ of bacteria suspensions after corresponding treatment.


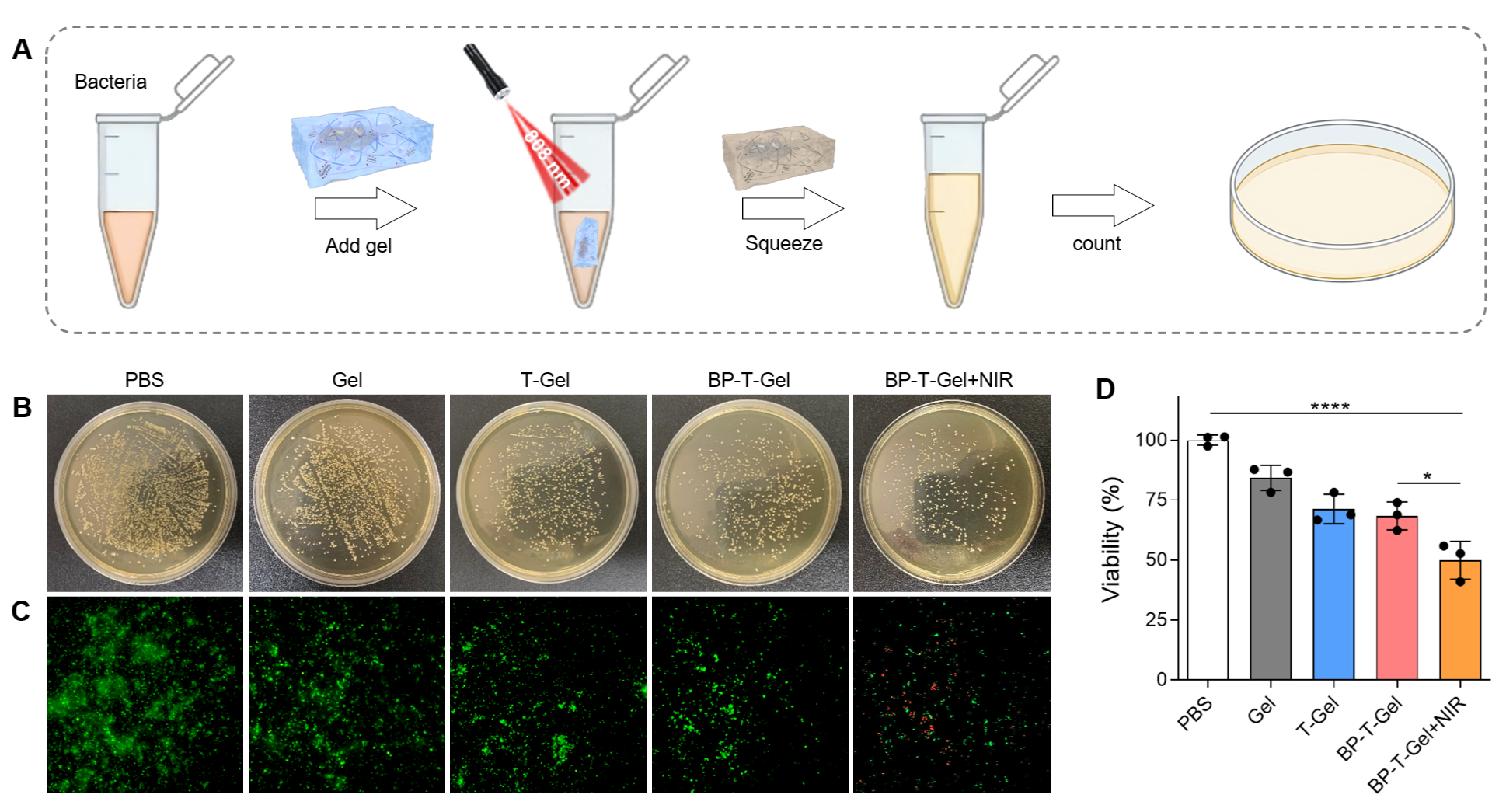


**Fig. S10.** (A) Schematic diagram of the antibacterial experiment process. (B) Represent photographs of agar plates and (C) corresponding colonies counts of MRSA treated with different samples. (D) Live/dead fluorescence images of MRSA treated with different samples. PI (red) was used to label dead bacteria and Syto-9 (green) to live bacteria. Data are presented as means ± SD (n = 3). *****p* < 0.0001. ***p* < 0.05. ns, no significant difference.


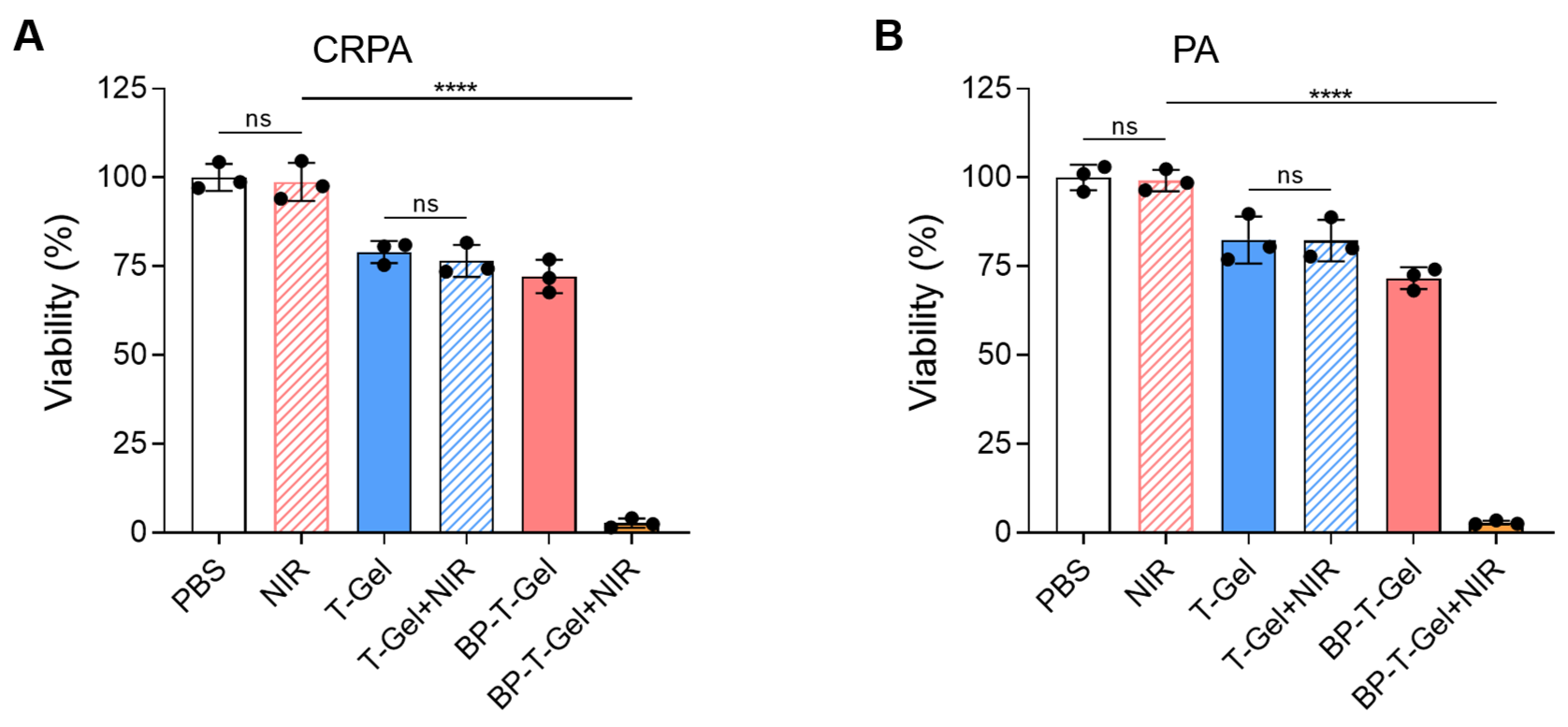


**Fig. S11.** Antibacterial performance of different samples against CRPA (A) and PA (B).


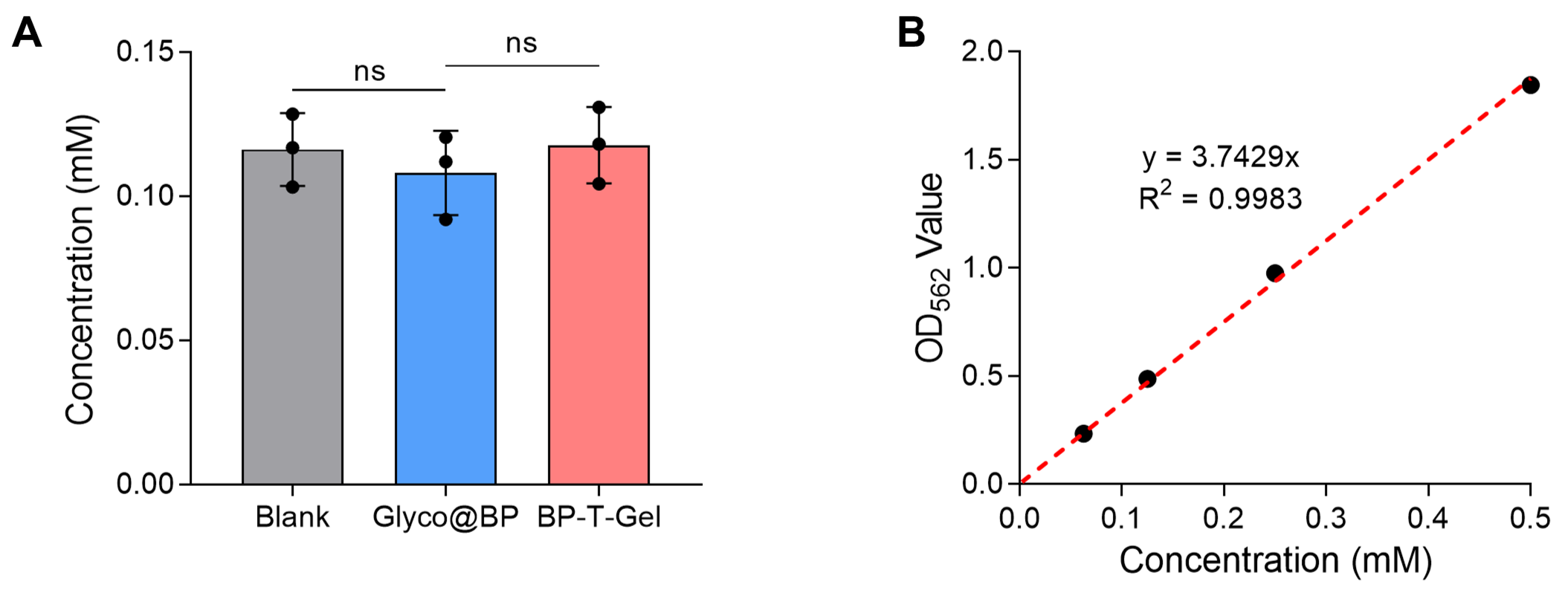


**Fig. S12.** (A) Calcium deposition in L929 cells. (B) Standard curve of calcium concentration induced by alizarin red S.


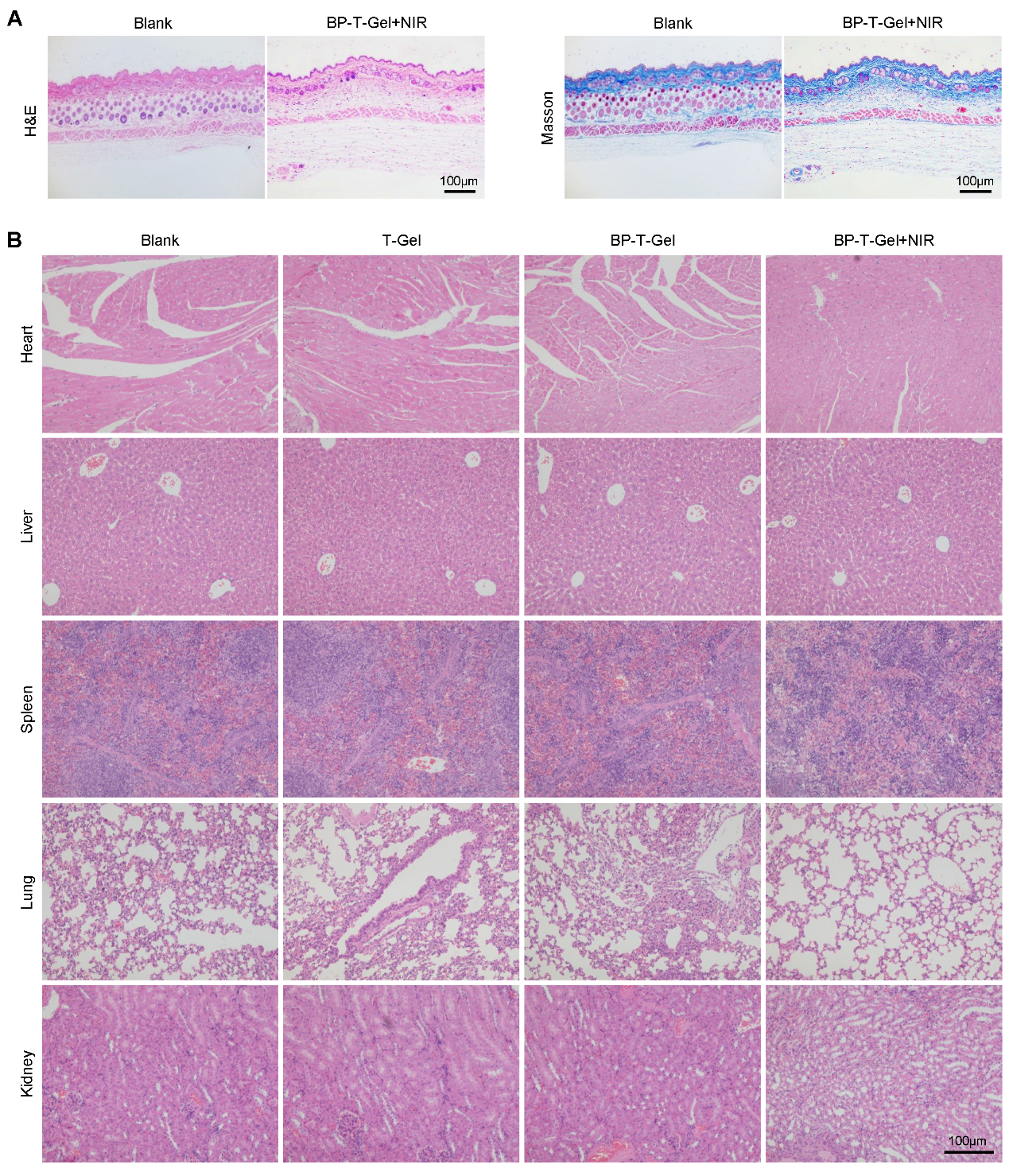


**Fig. S13.** (A) H&E and Masson staining of skin tissue from healthy mouse after BP-T-Gel+NIR treatment for 9 days. (B) H&E staining of main organs (heart, liver, spleen, lung, and kidney) from model mice after 9 days treatments.

**References**

[1] I. Kalograiaki, M. Abellán-Flos, L.Á. Fernández, M. Menéndez, S.P. Vincent, D. Solís, Direct Evaluation of Live Uropathogenic Escherichia coli Adhesion and Efficiency of Antiadhesive Compounds Using a Simple Microarray Approach, Analytical Chemistry 90(20) (2018) 12314-12321.

[2] H. Elferink, K. Geurts, S. Jue, S. MacCormick, G. Veeneman, T.J. Boltje, Synthesis and cellular uptake of carbamoylated mannose derivatives, Carbohydrate Research 481 (2019) 67-71.

[3] P. Jiang, L. Huang, J. Wang, Q. Li, H. Mu, Carboxymethyl chitosan-based multifunctional hydrogels incorporated with photothermal therapy against drug-resistant bacterial wound infection, International Journal of Biological Macromolecules 209 (2022) 452-463.

[4] D. Zhang, Y.K. Ren, Y.M. He, R. Chang, S. Guo, S.S. Ma, F.X. Guan, M.H. Yao, In situ forming and biocompatible hyaluronic acid hydrogel with reactive oxygen species-scavenging activity to improve traumatic brain injury repair by suppressing oxidative stress and neuroinflammation, Materials Today Bio 15 (2022).

[5] L. Huang, J. Wang, L. Kong, X. Wang, Q. Li, L. Zhang, J. Shi, J. Duan, H. Mu, ROS-responsive hyaluronic acid hydrogel for targeted delivery of probiotics to relieve colitis, International Journal of Biological Macromolecules 222 (2022) 1476-1486.

[6] J. Qu, X. Zhao, Y.P. Liang, T.L. Zhang, P.X. Ma, B.L. Guo, Antibacterial adhesive injectable hydrogels with rapid self-healing, extensibility and compressibility as wound dressing for joints skin wound healing, Biomaterials 183 (2018) 185-199.

[7] H. Qiu, F. Pu, Z. Liu, X. Liu, K. Dong, C. Liu, J. Ren, X. Qu, Hydrogel-based artificial enzyme for combating bacteria and accelerating wound healing, Nano Research 13(2) (2020) 496-502.

[8] Y.L. Jin, S.H. Liu, X.Y. Wang, C.Y. Wang, Q.F. Ruan, W. Li, Multifunctional Microneedle Patches Loaded With Engineered Nitric Oxide-Releasing Nanocarriers for Targeted and Synergistic Chronic Wound Therapy, Advanced Materials 37(5) (2025).

[9] Y. Zeng, C. Wang, J. Lei, X. Jiang, K. Lei, Y. Jin, T. Hao, W. Zhang, J. Huang, W. Li, Spatiotemporally responsive cascade bilayer microneedles integrating local glucose depletion and sustained nitric oxide release for accelerated diabetic wound healing, Acta Pharmaceutica Sinica B 14(11) (2024) 5037-5052.

[10] F. Wang, X. Wang, S. Li, Q. Yang, H. Mu, J. Li, Y. Yang, Chitosan and gelatin based sprayable hydrogels incorporating photothermal and long-acting antibiotic sterilization for infected wound management with shape adaptability, Carbohydrate Polymers 350 (2025) 123046.

[11] B. Shyla, Mahadevaiah, G. Nagendrappa, A simple spectrophotometric method for the determination of phosphate in soil, detergents, water, bone and food samples through the formation of phosphomolybdate complex followed by its reduction with thiourea, Spectrochimica Acta Part A: Molecular and Biomolecular Spectroscopy 78(1) (2011) 497-502.

[12] N. Verma, M. Singh, S. Kumari, K. Priya, In-silico studies of isolated biological active fractions of Vachellia nilotica leaves extract against MG 63 cell lines and biological applications, Biocatalysis and Agricultural Biotechnology 60 (2024).
